# Supplementary material for: Exploring nurses’ experiences of a tailored intervention to increase MMR vaccine acceptance in a Somali community in Stockholm, Sweden: a qualitative interview study
Source: BMJ Open. 2023 Feb 6;13(2):e067169. doi: 10.1136/bmjopen-2022-067169 (PMC9906253; doi:10.1136/bmjopen-2022-067169)
Supplement: Supplementary data [file bmjopen-2022-067169supp001.pdf]

Folkhälsomyndigheten
PUBLIC HEALTH AGENCY OF SWEDEN

TIP Sweden: Evaluation of tailored communication on vaccination in Rinkeby and Tensta, Stockholm

Interview Guide – Nurses at the Child Health Clinics

Aim: To investigate how the implemented interventions are experienced and received by CHC nurses and how the interventions have influenced their work towards vaccine-hesitant parents.

The interview

- Give a brief introduction on the purpose of the interview and procedures, as a follow-up to previous written information
- Consent to participate in the interview, recording, etc.

Date (yy/mm/dd):____/____/____

Code no. for the interview:

Place for the interview: _____________

Starting time: __:____ End: ___:____

Interviever: ____

Background information

- Employment title
- How long have you worked within the CHC/services?
- District you are working in (Rinkeby or Tensta)?
- How long have you worked in the district?

Your experience about the tailored interventions organized by the PHAS

- A series of three seminars was given during autumn in 2015, did you participate?
- Can you describe what you thought about the content of the seminars? What was good/less good?
- Did the seminars give you new knowledge? (About autism, cultural communication, summary about Wakefields research, diseases and the global situation?)
- Can you describe the discussions that followed the seminars?
- Have you been able to use the information from the lectures when you meet vaccine-hesitant parents? (Can you give an example of a recent conversation with a VH parent? what happened, what did they say, what was the outcome etc. How did they use the training? Perhaps ask for an example where the conversation went well, and one where it did not. ?)
- Would you like the seminars to be given again? (In what format? Themes?)
- Can you describe other interventions/competence enhancement activities related to vaccine hesitancy organized within the CHS? (What do you think about these? Have they been in useful, how?)

Solna Folkhälsomyndigheten, SE-171 82 Solna. Visit: Nobels väg 18. **Östersund** Folkhälsomyndigheten, SE-831 40 Östersund. Visit: Forskarens väg 3.
Telephone + 46 10 205 20 00 **E-mail** info@folkhalsomyndigheten.se **www.folkhalsomyndigheten.se**

Benefits from interventions by PHAS in your daily work

- Are you familiar with the other intervention activities within the project, targeting the Somali speaking parents? (What do you think about them? Somali film, seminars, peers, postcard)
- Have you had any use of the interventions, generally? Can you describe if / how you have benefited from these in your work? (Referred to the parents? Used for discussion))
- Do you know about the peer group created within the project? (Have you met anyone in the group? Collaborated in any way? Have they served as support in your work)

Changed mode of work following the intervention

- Has your mode of work changed or been affected since the time before the interventions started? (In what way? Regarding autism?)
- Do you as nurses work differently at the CHC when approaching vaccine-hesitant parents? (Can you describe how? Internal communication? Autism?)
- Can you identify other / new groups of parents in the neighborhood who are hesitant towards vaccinations?

Your perception on parents' experiences of the interventions and change in parents' knowledge

- How do you think that Somali speaking parents experience the interventions? (Have they been reached? Their attitude towards the activities, seminars, films, peers)
- What questions and concerns do the parents have today? (How do they reason?)
- Do you experience any differences in VH parent´s knowledge compared with the time before the intervention?
- Do you experience any differences in attitudes among vaccine-hesitant parents compared with the time before the intervention? (What differences? What about their perception of social norms around vaccination in the community? (social opportunity COM-B) Larger or minor problem?)
- How do parents reason about autism today? (Any changes? How?)
- Do you experience a difference in behavior (decision to vaccinate) among vaccine-hesitant parents as compared to how it was before the interventions? Do they perceive a difference in decision-making in parents (reflective versus automatic motivation COM-B)

Future perspectives

- Do you need any further support in specific questions/areas regarding vaccination? (Which areas? Format? Something you have missed? Other interventions?)
- Do you need further knowledge on specific issues related to vaccine-hesitancy? (Which? How would you like to have knowledge?)
- Do you have any suggestion on other ways to reach vaccine-hesitant parents (which the staff at the CHC can work with?
- Do you have any thoughts about the group of peers? (Any benefit from their work, suggestions for cooperation, joint activities)?

Before we finish the interview

- Is there anything else you would like to address or add that we have not yet discussed?

Thank you for your participation!

Solna Folkhälsomyndigheten, SE-171 82 Solna. Visit: Nobels väg 18. **Östersund** Folkhälsomyndigheten, SE-831 40 Östersund. Visit: Forskarens väg 3.
Telephone + 46 10 205 20 00 **E-mail** info@folkhalsomyndigheten.se **www.folkhalsomyndigheten.se**
