# Supplementary material for: Exploring nurses’ experiences of a tailored intervention to increase MMR vaccine acceptance in a Somali community in Stockholm, Sweden: a qualitative interview study
Source: BMJ Open. 2023 Feb 6;13(2):e067169. doi: 10.1136/bmjopen-2022-067169 (PMC9906253; doi:10.1136/bmjopen-2022-067169)
Supplement: Author's manuscript [file bmjopen-2022-067169.draft_revisions.pdf]

BMJ Open is committed to open peer review. As part of this commitment we make the peer review history of every article we publish publicly available.

When an article is published we post the peer reviewers' comments and the authors' responses online. We also post the versions of the paper that were used during peer review. These are the versions that the peer review comments apply to.

The versions of the paper that follow are the versions that were submitted during the peer review process. They are not the versions of record or the final published versions. They should not be cited or distributed as the published version of this manuscript.

BMJ Open is an open access journal and the full, final, typeset and author-corrected version of record of the manuscript is available on our site with no access controls, subscription charges or pay-per-view fees (http://bmjopen.bmj.com).

If you have any questions on BMJ Open's open peer review process please email info.bmjopen@bmj.com

BMJ Open

Exploring nurses' experiences of tailored interventions to increase MMR vaccine acceptance and uptake in a Somali community in Stockholm, Sweden.

Journal:	*BMJ Open*
Manuscript ID	bmjopen-2022-067169
Article Type:	Original research
Date Submitted by the Author:	05-Aug-2022

Complete List of Authors:	Appelqvist, Emma; Public Health Agency of Sweden, Department of Public Health Analysis and Data Management; Lund University, Clinical Microbiology, Department of Translational Medicine<br>Jama, Asha; Public Health Agency of Sweden, Department of Public Health Analysis and Data Management<br>Kulane, Asli; Karolinska Institutet, Department of Global Public Health<br>Roth, Adam; Lund University, Clinical Microbiology, Department of Translational Medicine; Public Health Agency of Sweden, Department of Public Health Analysis and Data Management<br>Lindstrand, Ann; World Health Organization, Department of Immunization, Vaccines and Biologicals, Unit Essential Programme on Immunization<br>Godoy-Ramirez, Karina; Public Health Agency of Sweden, The Office of the Head for Communicable Disease Control and Health Protection

Keywords:	Public health < INFECTIOUS DISEASES, Paediatric infectious disease & immunisation < PAEDIATRICS, PUBLIC HEALTH

SCHOLARONE™
Manuscripts

I, the Submitting Author has the right to grant and does grant on behalf of all authors of the Work (as defined in the below author licence), an exclusive licence and/or a non-exclusive licence for contributions from authors who are: i) UK Crown employees; ii) where BMJ has agreed a CC-BY licence shall apply, and/or iii) in accordance with the terms applicable for US Federal Government officers or employees acting as part of their official duties; on a worldwide, perpetual, irrevocable, royalty-free basis to BMJ Publishing Group Ltd ("BMJ") its licensees and where the relevant Journal is co-owned by BMJ to the co-owners of the Journal, to publish the Work in this journal and any other BMJ products and to exploit all rights, as set out in our licence.

The Submitting Author accepts and understands that any supply made under these terms is made by BMJ to the Submitting Author unless you are acting as an employee on behalf of your employer or a postgraduate student of an affiliated institution which is paying any applicable article publishing charge ("APC") for Open Access articles. Where the Submitting Author wishes to make the Work available on an Open Access basis (and intends to pay the relevant APC), the terms of reuse of such Open Access shall be governed by a Creative Commons licence – details of these licences and which Creative Commons licence will apply to this Work are set out in our licence referred to above.

Other than as permitted in any relevant BMJ Author's Self Archiving Policies, I confirm this Work has not been accepted for publication elsewhere, is not being considered for publication elsewhere and does not duplicate material already published. I confirm all authors consent to publication of this Work and authorise the granting of this licence.

For peer review only

Exploring nurses' experiences of tailored interventions to increase MMR vaccine acceptance and uptake in a Somali community in Stockholm, Sweden.

Authors; Emma Appelqvist, Asha Jama, Asli Kulane, Adam Roth, Ann Lindstrand and Karina Godoy-Ramirez.

Corresponding author
Emma Appelqvist, MPH
Affiliation: Public Health Agency of Sweden, Stockholm, Sweden
Department of Public Health Analysis and Data Management
Email: Emma.Appelqvist@folkhalsomyndigheten.se

Affiliation: Lund University, Lund, Sweden
Faculty of Medicine, Clinical Microbiology, Department of Translational Medicine

For peer review only

Abstract

Objectives

To explore nurse's experiences of a tailored intervention that supported them with knowledge and tools to use for their encounter and dialogue with parents having low vaccine acceptance.

Design

In-depth interviews were conducted with 11 nurses in November 2017 and analyzed using a thematic approach.

Setting

This study is part of a multi-component intervention project targeting nurses and Somali parents, implemented in 2015-2017 in Rinkeby and Tensta, Sweden, an area with lower MMR vaccination coverage. Research following the Tailoring Immunisation Programme (TIP) approach from WHO revealed that Somali parents in the community delayed MMR vaccination due to fear of autism despite lack of scientific evidence.

Participants

Nurses at child health centers involved in a tailored intervention. The intervention was implemented in 2015 and included a series of seminars, a narrative film, and an information card for distribution to parents.

Results

The qualitative analysis revealed an overarching theme of "*facilitation and improvement of nurses' encounter and dialogue regarding MMR vaccination with parents having vaccine concerns*". Two underlying themes were identified; "*Increased knowledge and confidence in conveying evidence-based information about autism and MMR vaccinations*" and *"specific usefulness of the tools to facilitate dialogue, dispel myth and reduce language barriers"*.

Conclusion

The tailored interventions targeting nurses facilitated and improved the nurse's encounter and dialogue regarding MMR vaccination with parents having vaccine concerns. Nurses increased their knowledge and confidence in conveying evidence-based information about autism and MMR vaccination and felt better equipped to both convey this information and dispel the myth in dialogue with the parents. Nurses have a crucial role in vaccine uptake and acceptance and interventions aiming to strengthen their communication with parents are therefore essential.

For peer review only - http://bmjopen.bmj.com/site/about/guidelines.xhtml

Strengths and limitations of this study

- Qualitative interviews and thematic analysis facilitated a deeper knowledge of nurse´s experiences of an intervention supporting encounter and dialogue with parents having low vaccine acceptance.
- In-depth interviews with nurses provided insights by thorough and rich data of their individual experiences.
- The results are relevant for the specific community and setting for the study, generalizing the findings to other settings should be done with caution.

For peer review only

For peer review only - http://bmjopen.bmj.com/site/about/guidelines.xhtml

Introduction

In Sweden, the vaccination coverage for measles, mumps, and rubella (MMR) vaccination has been high and stable the last decade and 97,1 percent of two-year-olds were vaccinated against MMR in 2020 (1). Cases of measles in Sweden have varied from 3 to 51 cases per year during the last ten years (2). All cases are either imported or epidemiologically linked to the imported cases. The Regional verification committee of WHO concluded measles and rubella to be eliminated in Sweden in 2015 and 2017, respectively (3). There are, however, regional and local pockets with lower MMR vaccination coverage. Rinkeby and Tensta are districts in the Region of Stockholm with a high percentage of residents with a foreign background of which approximately 30% are of Somali origin (4). There are two child health care centers (CHC) located in the area. In 2000 the vaccination coverage for MMR for two-year-olds in Rinkeby and Tensta started to decline, and between 2002 and 2013, the vaccination coverage has been stabilized around 70 percent. With the accumulation of susceptible unimmunized populations, the area had an increased risk of outbreaks. From January to March spring 2017, there was a measles outbreak in the area, including the Somali community. During the outbreak 12 cases of measles were reported, of which nine cases were children (5).

To strengthen the work to eliminate measles and rubella, the WHO Regional Office for Europe developed an approach, the Tailoring Immunization Programs (TIP), aiming to facilitate a deeper understanding of barriers and drivers for suboptimal vaccination coverage in particular communities (6). The TIP approach is based on scientific evidence from social and behavioral science research. In 2013, the Public Health of Sweden (PHAS) piloted the TIP approach focusing on the situational analysis and research phase in three communities with known or suspected suboptimal MMR vaccination coverage; anthroposophic, Somali, and undocumented migrants (7-10).

Results from the previous qualitative studies among parents in the Somali community showed that parents had low vaccine acceptance and delayed MMR vaccination primarily due to fear of that children would "stop talking" and develop autism (8). The concerns about vaccine-induced autism due to MMR vaccination originates from the spurious, retracted article published by Wakefield et al. in 1998 which suggested a relationship between MMR vaccine and autism (11). Although several extensive international studies have been conducted, no evidence for such a link between autism and MMR vaccines has been found (12, 13). Despite lack of evidence, the myth has been rooted among Somali parents in Rinkeby and Tensta for years. Similarly, lower vaccination coverage for MMR has also been observed in Somali communities in the US due to fear of autism and the link to the vaccine (14, 15). Parents in Rinkeby and Tensta would show up for all appointments at the preventative child health care centers (CHC) but only refrain from the 18 months appointment at which the MMR vaccine is offered and therefore access and convenience for vaccination services were not deemed to be a barrier for parents with low vaccine acceptance in the community as parents attended CHC services generally. It became evident that parents had a concern regarding MMR vaccination that was not properly addressed at the CHC. Previous research has highlighted the crucial role of nurses in decision-making about vaccinations for parents (16-19). Having an open and accepting approach is key in discussions regarding vaccinations (17, 18). Thus, there was a need for tailored interventions targeting both parents and nurses respectively in the area. As a result, multidimensional tailored interventions were designed and implemented in 2015 for two main target groups; Somali parents and CHC nurses in Rinkeby and Tensta.

This study is part of the evaluation of a multi-component intervention project implemented for parents and nurses in Rinkeby and Tensta. Our study aimed to explore nurse's experiences of a tailored intervention that supported them with knowledge and tools to use for their encounter and dialogue with parents having low vaccine acceptance.

For peer review only - http://bmjopen.bmj.com/site/about/guidelines.xhtml

Methods

Summary of the TIP-project in Rinkeby and Tensta, Stockholm

The national immunization program (NIP) in Sweden is voluntary and all vaccinations are offered free of charge. The first dose of MMR vaccination is offered at 18 months at CHCs and a second dose is offered by school health services in grades 1 and 2 (children aged 7 to 8 years). This study is part of an multi-component intervention project funded and implemented by the Public Health Agency of Sweden in Rinkeby and Tensta, Stockholm County, Sweden in 2015-2017 (20). The current study only focuses on the intervention activities for the nurses for which the aim was to increase the nurse's knowledge and their competence for providing objective information regarding MMR vaccinations in dialogue with parents having low vaccine acceptance. All nurses at CHC in both Rinkeby and Tensta were offered and accepted to participate in the intervention activities. The overall design and implementation of the intervention for both parents and nurses have been described in previously in detail (20).

The intervention activities for nurses included education and training as well as a narrative film with Somali role models and card to use as tools when interacting with parents. During three consecutive weeks in August and September 2015, all nurses were invited to a seminar series. Each seminar lasted 2 hours and covered different topics relating to MMR vaccination i.e measles, MMR vaccination, communication, and autism. The focus was particularly on the epidemiological situation and MMR vaccinations both in the context of Somalia, from where the parents originate, and in the context of Sweden where the parents live currently. In addition, an expert on autism presented the current knowledge of autism and its causes. How to use scientific evidence to debunk the link between autism and MMR vaccination, how to communicate with parents with low vaccine acceptance, and culturally tailor communication were also covered in the seminars. At the end of the third and final seminar, nurses attending the seminar ($n=12$) were asked to participate in a short survey with 11 questions for evaluation purposes. The result of the survey was used to inform the interview guide.

In addition, the intervention also included a 14 minute narrative film with Somali role models, entitled "Vaccination – a wise choice for your child" (20). The film included information about measles, MMR vaccination as well as experiences of parents, a nurse, and a religious leader. The film was in Somali with Swedish subtitles. The nurses were given a link to the film when it was published on YouTube in 2016. A two-sided card, one side in the Somali language and the other side in Swedish, was also given to the nurses as tools to use in their dialogues and to hand out to parents. The card included brief bullet points focusing on; vaccinations offered free of charge for all children, the importance of childhood vaccination to protect against infectious diseases, remember to vaccinate your child before traveling abroad. In addition, a link and QR-code to the narrative film and a website with further information were included on the card.

Qualitative interviews

Setting and participants

In the fall of 2017, all 12 nurses working at the CHC in Rinkeby and Tensta were invited to participate in qualitative in-depth interviews. The senior pediatrician at the Regional Preventive Child Health Services facilitated invitation to the study. Three of the nurses were employed after the seminar series in 2015 but they had been briefed and given the tools (film and card) used in the intervention, and hence they were invited to participate in the interviews. Participants were informed in advance about the purpose, design, and methodology of the study. All interviews were conducted in November 2017. In-depth interviews were held with 11 of the nurses who consented.

Data collection

The interviews were semi-structured using a thematic guide. The outline was adapted from a previous study (9). It also included questions regarding experiences of the interventions, benefits, and changes in daily work since the intervention as well as the perception of parents' experiences of the interventions. Nurses employed after the seminar series had been held were only asked questions of the parts of the tools in the intervention they had been able to use (film and card) and questions about the seminars were excluded. The interview guide was piloted by two members of the research team

For peer review only - http://bmjopen.bmj.com/site/about/guidelines.xhtml

with minor adaptions made. Interviews were conducted in Swedish by the two authors (EA, KGR) and digitally recorded. All interviews were held at the interviewee's workplace of their choice. The interviews ranged from 18 to 43 minutes, 34 minutes on average. Written informed consent was obtained before the start of each interview.

Data analysis
All interviews were transcribed verbatim and analysis was conducted using a thematic approach (21). The interviews were first read several times to become familiar with the content and thereafter coded by the first author. Two authors assessed and discussed initial coding for consistency after which themes were developed. Themes and initial coding were shared among the research team to elaborate and revise the themes until a final version was agreed upon. The analysis was conducted both manually and collected in Microsoft Office Excel.

Patient and public involvement
Patients and the public were not involved in the design of the study.

Ethical considerations
The study received ethical approval for the interviews from the Regional Ethics Committee in Stockholm, Sweden, Dnr 2016/1518-31/5.

Results
In-depth interviews were held with 11 nurses employed at the CHC in the fall of 2017. An overarching theme that emerged was: *facilitation and improvement of nurses' encounter and dialogue regarding MMR vaccination with parents having vaccine concerns.* And two underlying subthemes were identified;

- *Increased knowledge and confidence in conveying evidence-based information about autism and MMR vaccinations*
- *Specific usefulness of the tools to facilitate dialogue, dispel myth and reduce language barriers*.
    - Tailored seminars
    - The information card
    - The film

The results are presented in detail below with theme as bold and subthemes as italic and underlined headings. Important quotes from the interviews have been chosen to emphasize and support the results.

Facilitation and improvement of nurses' encounter and dialogue regarding MMR vaccination with parents having vaccine concerns

Increased knowledge and confidence in conveying evidence-based information about autism and MMR vaccination
The nurses expressed that they felt more confident in engaging in a dialogue with parents discussing MMR vaccination and autism after the intervention. Some nurses described being generally more secure in their professional role whereas other nurses explicitly described being more confident to engage in discussions due to increased knowledge.

> *"the more knowledge I have, the more confident I am to engage in discussions" [nurse, #1]*

Nurses described that there had been a change in the way they feel regarding discussing MMR vaccination and autism. After the intervention, they felt that they can say with confidence that there is no scientific evidence for the vaccine to cause autism whereas, before the intervention, some nurses expressed feeling insecure and hesitated to vaccinate the child if the parents had low vaccine acceptance. Instead of engaging in a dialogue in such a situation, some nurses just offered the possibility to postpone the MMR vaccination. As two nurses described it:

For peer review only - http://bmjopen.bmj.com/site/about/guidelines.xhtml

"what if I vaccinate and they get autism?" I was feeling guilty… now I know, it doesn't cause autism [nurse, #9]

"try to differentiate and say that autism is a separate thing, which I also can explain if you'd like, but the vaccine and the disease are not linked" [nurse, #8]

With reinforced and increased knowledge, nurses felt more secure and confident in saying that there is no scientific evidence for a link to autism, which in turn enabled a better discussion with parents. The dialogue between nurses and parents was facilitated as nurses now felt they could respond to the concerns of parents having low vaccine acceptance and not only describe the MMR vaccine but also confidently communicate the strong scientific evidence of the vaccine not causing autism. When parents brought up that children could become silent due to the vaccine, nurses could name autism and also had the knowledge to explain autism for the parents, elaborating the dialogue and responses to parents.

"feels safe to directly say that there is no evidence" [nurse, #4]

Although the nurses described positive changes, there were still challenges in the dialogue with parents. An issue raised was the delicate balance between discussing proactively versus trying to persuade the parents. In challenging discussions with parents, the nurses did not want to feel like they were a salesperson for the vaccine or persuading the parents in their vaccination decision instead of enabling the parents to make a voluntary decision themselves.

"I don't want to feel like a salesman when it's the most challenging" [nurse, #5]

Specific usefulness of the tools to facilitate dialogue, dispel myth and reduce language barriers.

Tailored seminars

The eight nurses taking part in the seminars were asked questions about their experiences in the interviews. Overall, the nurses expressed that the seminars were beneficial and valuable. The structure and format of the seminars were satisfactory and well received. Moreover, the nurses highlighted that the content and topics included were highly relevant and useful. Particularly, the nurses remembered the seminar about the epidemiological situation of measles in Somalia, the Somali vaccination program, and how parents in and from Somalia reason regarding vaccinations. They also remembered the seminar about autism. Gaining cultural insights as well as useful phrases to use in dialogue with parents were particular highlights from the seminar series. Moreover, nurses also emphasized that reinforcing that autism is a disorder with a complex etiology with a hereditary component, has helped them to describe autism to parents having low vaccine acceptance and also debunk the myth regarding the MMR vaccine.

Nurses felt they have been able to take the discussions with parents to another, deeper level after the seminars. They felt more knowledgeable and had practical tools for how to discuss MMR vaccinations and measles in a more structured way with the parents. Some highlighted that learning "one-liners" had been especially useful tools whereas others felt they had more evidence-based information as arguments.

"I received many tools for [use] when I came to work" [nurse, #4]

The nurses expressed that they had increased their knowledge and could dispel the myth of a link between MMR vaccination and autism in their dialogue and discussion with parents. They emphasized that it was very valuable to learn more about the scientific evidence and large epidemiological studies that have been conducted regarding MMR vaccinations and autism. Knowing the thorough research and the current solid evidence base made the nurses feel more confident, not only in their own beliefs but also in their conversations and in conveying the information to the parents. The nurses found new ways to approach and motivate the parents and were able to handle the discussion differently when parents had low vaccine acceptance and worried about autism and MMR vaccination. In addition, nurses felt more knowledgeable regarding autism, its early signs and symptoms, and the complexity of factors potentially involved in the onset of disease, despite the etiology not yet being unclear.

For peer review only - http://bmjopen.bmj.com/site/about/guidelines.xhtml

"had the greatest benefit of being able to separate and explain what autism is and what the vaccine is, in my dialogues with parents" [nurse, #6]

"I have found good arguments based on research and [I] can confidently tell it to others, indeed" [nurse, #10]

<u>Information card</u>

Nurses described the usefulness of the card and how it had been helpful to hand it out directly to the parents. They particularly highlighted the link to the film and recommended parents to watch the film at home. Moreover, a stack of cards could lay on the nurses' desk, making it highly visible and easier to remember to hand it out. By handing out the card with information in both Swedish and Somali, the nurses knew exactly the content of the information they gave to the parents directly. In cases of relying on interpreters, some of the nurses were concerned that some of the interpreters may not know how to translate fully the information regarding the diseases and vaccinations correctly and hence the parents may not have received all the information. Thus, by using the card the nurses were ensured the parents received the essential and correct information.

"[it has] been really good to hand something over and ask them to watch the film and then come back" [nurse, #9]

<u>The film</u>

Nurses perceived the film positively, particularly since it was a visual tool and available in the Somali language, decreasing language barriers and facilitating a better understanding of the content. The nurses had different ways of using the film. Many nurses recommended parents to watch the film at home with their spouse and also other family members, relatives, or friends, and then they would discuss any questions or concerns raised during the next appointment at CHC. The information card containing the link to the film was often given as a way to promote the film. Other nurses watched the film together with the parents during consultations. Some nurses thought that the film was too long to watch together with parents during appointments as they have a lot to cover during the appointments and not enough time to show a film of 15 minutes. At one of the CHCs, the film was shown on repeat in the waiting room so that parents could watch while waiting for their appointment. On occasions, nurses could sit and watch the film together with parents in the waiting room although some nurses thought it was not an optimal approach as they believed they might single out parents as having low vaccine acceptance.

Nurses emphasized that the film helped parents to reconsider their decision for MMR vaccination as a few nurses recalled and gave examples of situations where parents with low vaccine acceptance had changed their mind after watching the movie and chose to vaccinate their child against MMR. Other parents may not have changed their decision regarding MMR vaccination, but the nurses felt that they were less sceptic towards the vaccine.

"came back to vaccinate after having watched the film" [nurse, #1]

"some become softer, the film is really helpful actually" [nurse, #4]

According to the nurses, the parents believed the film was a valuable tool because it included relevant evidence-based information and highlighted the perspective of the parents. Moreover, the film was perceived as credible as most of the experts and parents participating in the film were of Somali origin and spoke Somali. The experts were also well-known, trusted, and respected authorities in the community.

Nurses expressed that they perceived parents to be less questioning and more open for discussions related to MMR vaccination after the interventions. Parents had become more open to receiving information and having a dialogue. Moreover, the nurses also felt that parents had gained more knowledge, in particular about autism and posed less questions about the disease.

"[it is] difficult to measure, it is just a feeling I have" [nurse, #1]

"[I] believe that there are more who are knowledgeable about autism, actually" [nurse, #8]

For peer review only - http://bmjopen.bmj.com/site/about/guidelines.xhtml

In addition, the card and the film had been found to be especially useful tools during the outbreak of measles after the intervention, as expressed by this nurse:

"During the outbreak we tried to reach out in every way by using information cards, the film, visit to open day care, pediatricians informing…" [nurse, #7]

Discussion

The present study indicates that tailored interventions with seminars and tools for nurses can facilitate and enhance the dialogue with parents of Somali origin in Tensta and Rinkeby having low vaccine acceptance. The tailored intervention supported the dialogue, dispelled myth and reduced language barriers while also gave nurses a better understanding of the parental perspective and cultural insights concerning MMR vaccination and reasons for the parents' concerns. Importantly, nurses gained additional knowledge and confidence in the scientific evidence supporting that MMR vaccination does not cause autism and they were now able to dispel the myth in their dialogue and better convey this information to the parents. By using the tools, knowledge, and improved understanding of the parent's perspective, the nurses engaged in discussions regarding MMR vaccinations and autism with parents in a different way. The seminars were of importance for both providing and reinforcing knowledge to boost the nurses' confidence in discussing autism which made them feel more secure when communicating with the parents. In addition, tools were also useful during the outbreak of measles in 2017.

Before the intervention was implemented, key barriers were parents fearing that their children would "stop talking" following an MMR vaccination and some parents also described unpleasant encounters with nurses (8, 9). On the other hand, a key facilitator for vaccinating parents in the community was their trust in nurses (8, 9). Autism was a sensitive topic that both parents and nurses sidestepped when discussing MMR vaccinations. Similar barriers for vaccination due to fear of autism has also been observed in a Somali community in the US (14, 15).

Globally, there was an increase of measles cases in Europe in 2018-2019, although the disease could be prevented and even eradicated by effective vaccines. Several of the countries with a high incidence of measles have a vaccination coverage well below the recommended threshold for herd immunity. Factors influencing a low, sub-optimal, vaccination coverage are context specific, complex, and multidimensional (22). Factors affecting declining vaccine acceptance include barriers in availability and convenience in health care systems as well as social determinants, social and cultural norms (23). In addition, these factors are often specific for their context, setting, type of vaccine and they may also change across time (22). Limited access to health care and vaccination services for populations has influenced sub-optimal vaccination coverage in Europe (24).

Using the TIP framework for this intervention project was useful for gaining a thorough understanding of the barriers and drivers for MMR vaccination in the Somali community (20). Previous studies have highlighted the important and crucial role of nurses and health care providers for parental vaccine acceptance, trust, and decision making (25-28). The belief and attitudes of health care workers are essential to parental vaccine acceptance and the demand for childhood vaccinations (29, 30). Hence, it was deemed necessary to tailor interventions for the nurses specifically as part of the multidimensional intervention project and to complement the interventions targeting the parents. Multicomponent, dialogue-based, and tailored interventions appear to be more effective.(31). In addition, an important aspect of the Swedish national immunization program is the Child Healthcare Services structure and its responsibility for providing and implementing childhood vaccinations (32). Parents often meet the same nurses for the child health and development checks as well for vaccinations and therefore the nurses have a crucial role in the success of the vaccination program. Although our present study suggests improvements in the dialogue, the nurses will still be faced with recurring challenges and parents having questions and concerns regarding vaccinations. Hence, continued work and effort are needed to support the positive trend in this community. The current study assessed the dialogue and situation 2 years after the implementation of the intervention. Further evaluations of long-term outcomes are needed as well as assessment of the vaccination coverage in the community over time.

For peer review only - http://bmjopen.bmj.com/site/about/guidelines.xhtml

Based on the results of our current study, it would be important that newly recruited nurses in the community are offered similar training and introduction to the tools. The current intervention was implemented 2015-2017, repeating the seminars and reinforcing the information for all nurses may also be important for continued capacity building and sustainability.

Evaluations of tailored interventions are needed to increase the evidence base for effective interventions that can be used by public health authorities to not only increase vaccination acceptance and uptake but also contribute to resilience for national immunization programs. By addressing pockets with suboptimal vaccination coverage and identifying the facilitators and barriers to vaccination, interventions can be designed and implemented to improve vaccination uptake and health equity.

Methodological considerations

Throughout the research process, trustworthiness has been strengthened by several activities. All interviews were conducted in a private space chosen by the interviewee to allow for openhearted discussions. Several members of the interdisciplinary research team have expertise in qualitative methodology and have also become familiar with the setting, community, and culture over an extended period. Moreover, the research team familiarized themselves with the data initially and has regularly discussed the analysis for strengthening validity throughout. This study is limited, however, as it only focused on the perspective of the nurses, further studies are needed to capture the perspective of the parents. Three of the nurses were employed after the seminar series in 2015 but had the possibility to use the information card and the film. Thus they missed part of the intervention and didn't have the same extended time using the tools as the nurses who were employed before the start of the intervention. Although the number of participants in this interview study is small the results are relevant for this specific community and setting, generalizing the findings to other settings should be done with caution.

Conclusion

Following the implementation of tailored interventions targeting nurses working in an area with low MMR vaccination coverage, the nurse's dialogue was facilitated and enhanced with parents' vaccination concerns. Particularly, nurses increased their knowledge and confidence in conveying evidence-based information about autism and MMR vaccination and felt better equipped to convey this information to the parents and to dispel the myth linking autism and MMR in their dialogue with the parents. In addition, the tailored tools also helped to reduce language barriers. Child health nurses have a crucial role in the vaccine acceptance and uptake in the Swedish national immunization program and interventions aiming to strengthen the nurses in their communication and dialogue with parents are therefore important. Further studies are needed for assessing long-term outcomes of the multi-component intervention as well as vaccination coverage within the studied community.

Acknowledgments

The authors wish to thank the Stockholm Region child health services for their support and collaboration for this study. A sincere thank you to all the nurses at the child health care centers who kindly participated with openness and commitment.

Contributors

EA, AJ, AK, AL and KGR designed the study. All authors contributed to the analysis and interpretation of data. EA, AJ and KGR drafted the first version of this paper and AK, AL and AR have revised the paper critically and substantially. The final version of the paper has been approved by all authors and they all agree to be accountable for all aspects of the work.

Funding

The current study was funded by The Public Health Agency of Sweden.

For peer review only - http://bmjopen.bmj.com/site/about/guidelines.xhtml

Conflict of interest

The authors declare no conflict of interest.

Ethical approval

The study received ethical approval for the interviews from the Regional Ethics Committee in Stockholm, Sweden, Dnr 2016/1518-31/5.

Data Sharing Statement

No data are available.

For peer review only

For peer review only - http://bmjopen.bmj.com/site/about/guidelines.xhtml

References

1. Folkhälsomyndigheten. Barnvaccinationsprogrammet i Sverige 2020 - Årsrapport. 2021.
2. Folkhälsomyndigheten. Barnvaccinationsprogrammet i Sverige 2019 – Årsrapport. 2020.
3. World Health Organization. SIXTH MEETING OF THE EUROPEAN REGIONAL VERIFICATION COMMISSION FOR MEASLES AND RUBELLA ELIMINATION (RVC). 2017.
4. Folkhälsomyndigheten. Barriers and motivating factors to MMR vaccination in communities with low coverage in Sweden. 2015.
5. Folkhälsomyndigheten. Barnvaccinationsprogrammet i Sverige 2017. 2018.
6. World Health Organization. Tailoring Immunization Programmes (TIP). Copenhagen: WHO Regional Office for Europe; 2019.
7. Bystrom E, Lindstrand A, Likhite N, Butler R, Emmelin M. Parental attitudes and decision-making regarding MMR vaccination in an anthroposophic community in Sweden--a qualitative study. Vaccine. 2014;32(50):6752-7.
8. Jama A, Ali M, Lindstrand A, Butler R, Kulane A. Perspectives on the Measles, Mumps and Rubella Vaccination among Somali Mothers in Stockholm. International journal of environmental research and public health. 2018;15(11).
9. Jama A, Lindstrand A, Ali M, Butler R, Kulane A. Nurses' Perceptions Of MMR Vaccine Hesitancy In An Area With Low Vaccination Coverage. Pediatric health, medicine and therapeutics. 2019;10:177-82.
10. Godoy-Ramirez K, Byström E, Lindstrand A, Butler R, Ascher H, Kulane A. Exploring childhood immunization among undocumented migrants in Sweden - following qualitative study and the World Health Organizations Guide to Tailoring Immunization Programmes (TIP). Public health. 2019;171:97-105.
11. Wakefield AJ, Murch SH, Anthony A, Linnell J, Casson DM, Malik M, et al. Ileal-lymphoid-nodular hyperplasia, non-specific colitis, and pervasive developmental disorder in children. Lancet. 1998;351(9103):637-41.
12. Taylor LE, Swerdfeger AL, Eslick GD. Vaccines are not associated with autism: an evidence-based meta-analysis of case-control and cohort studies. Vaccine. 2014;32(29):3623-9.
13. Madsen KM, Hviid A, Vestergaard M, Schendel D, Wohlfahrt J, Thorsen P, et al. A population-based study of measles, mumps, and rubella vaccination and autism. The New England journal of medicine. 2002;347(19):1477-82.
14. Bahta L, Ashkir A. Addressing MMR Vaccine Resistance in Minnesota's Somali Community. Minnesota medicine. 2015;98(10):33-6.
15. Gahr P, DeVries AS, Wallace G, Miller C, Kenyon C, Sweet K, et al. An Outbreak of Measles in an Undervaccinated Community. Pediatrics. 2014;134(1):e220-e8.
16. Paterson P, Meurice F, Stanberry LR, Glismann S, Rosenthal SL, Larson HJ. Vaccine hesitancy and healthcare providers. Vaccine. 2016;34(52):6700-6.
17. Ames HM, Glenton C, Lewin S. Parents' and informal caregivers' views and experiences of communication about routine childhood vaccination: a synthesis of qualitative evidence. Cochrane Database Syst Rev. 2017;2(2):Cd011787.
18. Berry NJ, Danchin M, Trevena L, Witteman HO, Kinnersley P, Snelling T, et al. Sharing knowledge about immunisation (SKAI): An exploration of parents' communication needs to inform development of a clinical communication support intervention. Vaccine. 2018;36(44):6480-90.
19. Karafillakis E, Dinca I, Apfel F, Cecconi S, Wűrz A, Takacs J, et al. Vaccine hesitancy among healthcare workers in Europe: A qualitative study. Vaccine. 2016;34(41):5013-20.
20. Jama A, Appelqvist E, Kulane A, Karregård S, Rubin J, Nejat S, et al. Design and implementation of tailored intervention to increase vaccine acceptance in a Somali community in Stockholm, Sweden - based on the Tailoring Immunizations Programs approach. Accepted. 2022.
21. Braun V, Clarke V. Using thematic analysis in psychology. Qualitative Research in Psychology. 2006;3(2):77-101.
22. MacDonald NE. Vaccine hesitancy: Definition, scope and determinants. Vaccine. 2015;33(34):4161-4.

For peer review only - http://bmjopen.bmj.com/site/about/guidelines.xhtml

23. Dubé E, Gagnon D, MacDonald N, Bocquier A, Peretti-Watel P, Verger P. Underlying factors impacting vaccine hesitancy in high income countries: a review of qualitative studies. Expert Review of Vaccines. 2018;17(11):989-1004.
24. Boyce T, Gudorf A, de Kat C, Muscat M, Butler R, Habersaat KB. Towards equity in immunisation. Euro Surveill. 2019;24(2).
25. Leask J, Kinnersley P, Jackson C, Cheater F, Bedford H, Rowles G. Communicating with parents about vaccination: a framework for health professionals. BMC pediatrics. 2012;12:154.
26. Gust DA, Darling N, Kennedy A, Schwartz B. Parents with doubts about vaccines: which vaccines and reasons why. Pediatrics. 2008;122(4):718-25.
27. Glanz JM, Wagner NM, Narwaney KJ, Shoup JA, McClure DL, McCormick EV, et al. A mixed methods study of parental vaccine decision making and parent-provider trust. Academic pediatrics. 2013;13(5):481-8.
28. Cooper LZ, Larson HJ, Katz SL. Protecting public trust in immunization. Pediatrics. 2008;122(1):149-53.
29. Simone B, Carrillo-Santisteve P, Lopalco PL. Healthcare workers role in keeping MMR vaccination uptake high in Europe: a review of evidence. Euro surveillance : bulletin europeen sur les maladies transmissibles = European communicable disease bulletin. 2012;17(26):20206.
30. Dube E. Addressing vaccine hesitancy: the crucial role of healthcare providers. Clin Microbiol Infect. 2017;23(5):279-80.
31. Dubé E, Gagnon D, MacDonald NE. Strategies intended to address vaccine hesitancy: Review of published reviews. Vaccine. 2015;33(34):4191-203.
32. Wettergren B, Blennow M, Hjern A, Söder O, Ludvigsson JF. Child Health Systems in Sweden. The Journal of Pediatrics. 2016;177:S187-S202.

For peer review only

BMJ Open

Exploring nurses' experiences of a tailored intervention to increase MMR vaccine acceptance in a Somali community in Stockholm, Sweden: A qualitative interview study

Journal:	*BMJ Open*
Manuscript ID	bmjopen-2022-067169.R1
Article Type:	Original research
Date Submitted by the Author:	23-Dec-2022
Complete List of Authors:	Appelqvist, Emma; Public Health Agency of Sweden, Department of Public Health Analysis and Data Management; Lund University, Clinical Microbiology, Department of Translational Medicine<br>Jama, Asha; Public Health Agency of Sweden, Department of Public Health Analysis and Data Management<br>Kulane, Asli; Karolinska Institutet, Department of Global Public Health<br>Roth, Adam; Lund University, Clinical Microbiology, Department of Translational Medicine; Public Health Agency of Sweden, Department of Public Health Analysis and Data Management<br>Lindstrand, Ann; World Health Organization, Department of Immunization, Vaccines and Biologicals, Unit Essential Programme on Immunization<br>Godoy-Ramirez, Karina; Public Health Agency of Sweden, The Office of the Head for Communicable Disease Control and Health Protection
**Primary Subject Heading**:	Public health
Secondary Subject Heading:	Qualitative research
Keywords:	Public health < INFECTIOUS DISEASES, Paediatric infectious disease & immunisation < PAEDIATRICS, PUBLIC HEALTH

SCHOLARONE™
Manuscripts

I, the Submitting Author has the right to grant and does grant on behalf of all authors of the Work (as defined in the below author licence), an exclusive licence and/or a non-exclusive licence for contributions from authors who are: i) UK Crown employees; ii) where BMJ has agreed a CC-BY licence shall apply, and/or iii) in accordance with the terms applicable for US Federal Government officers or employees acting as part of their official duties; on a worldwide, perpetual, irrevocable, royalty-free basis to BMJ Publishing Group Ltd ("BMJ") its licensees and where the relevant Journal is co-owned by BMJ to the co-owners of the Journal, to publish the Work in this journal and any other BMJ products and to exploit all rights, as set out in our licence.

The Submitting Author accepts and understands that any supply made under these terms is made by BMJ to the Submitting Author unless you are acting as an employee on behalf of your employer or a postgraduate student of an affiliated institution which is paying any applicable article publishing charge ("APC") for Open Access articles. Where the Submitting Author wishes to make the Work available on an Open Access basis (and intends to pay the relevant APC), the terms of reuse of such Open Access shall be governed by a Creative Commons licence – details of these licences and which Creative Commons licence will apply to this Work are set out in our licence referred to above.

Other than as permitted in any relevant BMJ Author's Self Archiving Policies, I confirm this Work has not been accepted for publication elsewhere, is not being considered for publication elsewhere and does not duplicate material already published. I confirm all authors consent to publication of this Work and authorise the granting of this licence.

For peer review only

Exploring nurses' experiences of a tailored intervention to increase MMR vaccine acceptance in a Somali community in Stockholm, Sweden: A qualitative interview study

Authors; Emma Appelqvist, Asha Jama, Asli Kulane, Adam Roth, Ann Lindstrand och Karina Godoy-Ramirez.

word count: 4550 words
abstract count: 237 words
number of references: 38 references

Corresponding author

Emma Appelqvist, MPH
Affiliation: Public Health Agency of Sweden, Stockholm, Sweden
Department of Public Health Analysis and Data Management
Address: Nobels väg 18, SE-171 82 Solna, Sweden
Tel.: +46 (0)10 205 2022, Email: Emma.Appelqvist@folkhalsomyndigheten.se

Affiliation: Lund University, Lund, Sweden
Faculty of Medicine, Clinical Microbiology, Department of Translational Medicine
Address: Jan Waldenströms gata 59, 205 02 Malmö, Sweden

Co-authors

Asha Jama, MPH
Affiliation: Public Health Agency of Sweden, Stockholm, Sweden
Department of Public Health Analysis and Data Management
Address: Nobels väg 18, SE-171 82 Solna, Sweden
Tel.: +46 (0)10 205 2082, Email: Asha.Jama@folkhalsomyndigheten.se

Asli Kulane, MD, MSc, PhD, Associate Professor
Affiliation: Karolinska Institutet, Stockholm, Sweden
Department of Global Public Health
Address: Tomtebodavägen 18A, SE-171 77 Stockholm, Sweden
Tel.: +46 (0) 8-524 833 88, Email: Asli.Kulane@ki.se

Adam Roth, MD, PhD, Associate Professor
Affiliation: Public Health Agency of Sweden, Stockholm, Sweden
Department of Public Health Analysis and Data Management
Address: Nobels väg 18, SE-171 82 Solna, Sweden
Email; adam.roth@med.lu.se

Affiliation: Lund University, Lund, Sweden
Faculty of Medicine, Clinical Microbiology, Department of Translational Medicine
Address: Jan Waldenströms gata 59, 205 02 Malmö, Sweden

Ann Lindstrand, MD, MPH, PhD
Affiliation: World Health Organization (WHO) Headquarters, Geneva, Switzerland
Department of Immunization, Vaccines and Biologicals, Unit Essential Programme on Immunization
Address: 20 Avenue Appia, 1211 Geneva 27, Switzerland
Tel.: +41 227913436, Email: lindstranda@who.int

Karina Godoy-Ramirez, MSc, MPH, PhD
Affiliation: Public Health Agency of Sweden, Stockholm, Sweden
The Office of the Head for Communicable Disease Control and Health Protection
Address: Nobels väg 18, SE-171 82 Solna, Sweden
Tel.: +46 (0)10 205 2668, Email: Karina.Godoy@folkhalsomyndigheten.se

For peer review only - http://bmjopen.bmj.com/site/about/guidelines.xhtml

Exploring nurses' experiences of a tailored intervention to increase MMR vaccine acceptance in a Somali community in Stockholm, Sweden: A qualitative interview study

Authors; Emma Appelqvist, Asha Jama, Asli Kulane, Adam Roth, Ann Lindstrand and Karina Godoy-Ramirez.

Corresponding author
Emma Appelqvist, MPH
Affiliation: Public Health Agency of Sweden, Stockholm, Sweden
Department of Public Health Analysis and Data Management
Email: Emma.Appelqvist@folkhalsomyndigheten.se

Affiliation: Lund University, Lund, Sweden
Faculty of Medicine, Clinical Microbiology, Department of Translational Medicine

Abstract

Objectives
To explore nurses' experiences of a tailored intervention that supported them with knowledge and tools to use during their encounters and dialogue with parents having low vaccine acceptance.

Design
A qualitative study with in-depth interviews conducted in 2017. Data were analyzed using thematic analysis.

Setting
This study was part of a multi-component intervention targeting Somali parents and the nurses at child health centers in the Rinkeby and Tensta neighborhoods of Stockholm. An area with documented low MMR vaccination coverage. Previous research has revealed that Somali parents in the community delayed MMR vaccination due to fear of autism despite lack of scientific evidence. The interventions were implemented in 2015-2017.

Participants
11 nurses employed at the child health centers involved in the intervention participated in interviews. The tailored intervention targeting nurses included a series of seminars, a narrative film, and an information card with key messages for distribution to parents.

Results
The qualitative analysis revealed an overarching theme: perception of improved communication with parents. Two underlying themes were identified (1) feeling more confident to address parents' MMR vaccine concerns (2) diverse tools as useful support to dispel myth and reduce language barriers.

Conclusion
From the nurses' perspective, the tailored intervention was useful to improve communication with parents having vaccine concerns. Nurses have a crucial role in vaccine uptake and acceptance. Interventions aiming to strengthen their communication with parents are therefore essential, especially in areas with lower vaccine acceptance.

Strengths and limitations of this study

- Qualitative interviews and thematic analysis enabled a deeper understanding of nurses' experiences of a tailored intervention supporting encounters and dialogue with parents having low vaccine acceptance.

For peer review only - http://bmjopen.bmj.com/site/about/guidelines.xhtml

- In-depth interviews of nurses provided unique insights into their experiences of using tailored tools.
- This study is limited as it only focused on the perspective of the nurses', further studies are needed to capture the perspective of the parents.
- The results are relevant for the specific community and setting for the study, generalizing the findings to other settings should be done with caution.

For peer review only

Introduction

Cases of measles increased in Europe and the WHO European Region in 2017-2019, although the disease could be prevented and even eradicated by effective vaccines [1, 2]. In Europe alone, 14 600 cases were reported in 2017 and in the WHO European Region more than 82 000 cases were reported [1, 2]. The majority of the cases occurred among under-immunized individuals and in pockets of lower vaccination coverage below the recommended threshold of 95% coverage for achieving herd immunity [1, 2]. The measles, mumps, rubella (MMR) vaccination coverage has been high and stable throughout the last decade in Sweden, where 97.1 % of two-year children were vaccinated against MMR in 2020 [3]. Cases of measles in Sweden have varied from 3 to 51 cases per year during the last ten years [4]. All recent cases have either been imported or epidemiologically linked to an imported case, apart from a few sporadic cases of unknown origin [personal communication, Hélène Englund, The Public Health Agency of Sweden, 2022-11-15]. The Regional verification committee of WHO concluded measles and rubella to be eliminated in Sweden in 2015 and 2017, respectively [5]. There are, however, regional and local pockets with lower MMR vaccination coverage. The national immunization program (NIP) in Sweden is voluntary and all vaccinations are offered free of charge. The first dose of MMR vaccination is offered at 18 months at child health care centers (CHC) and a second dose is offered by school health services in grades 1 and 2 (children aged 7 to 8 years). Rinkeby and Tensta are districts in the Region of Stockholm with a high percentage of residents with a foreign background of which approximately 30% are of Somali origin [6]. There are two CHCs located in the area. In 2000 the vaccination coverage for MMR for two-year-olds in Rinkeby and Tensta started to decline, and between 2002 and 2013, the vaccination coverage has been stabilized around 70 %. With the accumulation of susceptible unimmunized populations, the area had an increased risk of measles outbreaks. From January to March spring 2017, there was a measles outbreak in the area, which afflicted the Somali community. During the outbreak 12 cases of measles were reported, of which nine cases were children [7].

To strengthen the work to eliminate measles and rubella, the WHO Regional Office for Europe developed an approach, the Tailoring Immunization Programs (TIP), aiming to facilitate a deeper understanding of barriers and drivers for suboptimal vaccination coverage in particular communities [8]. Based on findings of barriers and drivers, tailored and targeted interventions may be designed and implemented to support improved and equitable vaccination uptake [9]. The TIP approach is based on scientific evidence from social and behavioral science research, underpinned by the Capability-Opportunity-Motivation-Behaviour (COM-B) model and the Behaviour Change Wheel (BCW) framework which has been adapted for behavioral insights for vaccinations [8, 10]. In 2013, the Public Health Agency of Sweden (PHAS) piloted the TIP approach and conducted formative research (TIP phase 1-2, based on the TIP Guide version 1, 2013) in three communities with known or suspected suboptimal MMR vaccination coverage; anthroposophic, Somali, and undocumented migrants [11-14].

Summary of the TIP-project in Rinkeby and Tensta, Stockholm

Results from the previous qualitative studies among parents in the Somali community showed that parents had low vaccine acceptance and delayed MMR vaccination primarily due to fear that children would “stop talking“ and develop autism [12]. The concerns about vaccine-induced autism due to MMR vaccination originate from the spurious, retracted article published by Wakefield et al. in 1998 which suggested a link between the MMR vaccine and autism [15]. Although several extensive international studies have been conducted, no evidence for such a link between autism and MMR vaccines has been found [16, 17]. Despite the lack of evidence, the myth has been rooted among Somali parents in Rinkeby and Tensta for years [12]. Similarly, lower vaccination coverage for MMR has also been observed in Somali communities in the US due to fear of autism and the link to the vaccine [18, 19]. Previous studies showed that parents in Rinkeby and Tensta would attend all appointments at the CHC but only refrain from the 18 months appointment at which the MMR vaccine is offered. Thus, access and convenience for vaccination services were not deemed to be a barrier for parents with low vaccine acceptance in the community as parents attended CHC generally. Moreover, it was evident that nurses did not address parents having a concern regarding MMR vaccination at the CHC [13] Previous research has highlighted the crucial role of nurses in decision-making about vaccinations for parents [20-23]. Having an open and accepting approach is key in discussions

For peer review only - http://bmjopen.bmj.com/site/about/guidelines.xhtml

regarding vaccinations [21, 22]. As a result, multi-component tailored interventions were designed and implemented in 2015-2017 for two main target groups in Rinkeby and Tensta; Somali parents and CHC nurses. The design and implementation of the intervention have been described in detail previously [24]. Although the intervention project had two main target groups, this study only focuses on the evaluation of the intervention activities for the nurses. Our current study aimed to explore nurses' experiences of a tailored intervention that supported them with knowledge and tools to use during their encounters and dialogue with parents having low vaccine acceptance.

For peer review only

For peer review only - http://bmjopen.bmj.com/site/about/guidelines.xhtml

Methods

Description of intervention activities and tools

The intervention activities for nurses included education and training as well as a narrative film with Somali role models and information card to use as tools when interacting with parents. The overall design and implementation of the intervention for both parents and nurses have been described in previously published research [24]. All 12 nurses working at the CHCs in either Rinkeby or Tensta at the time of implementation were offered and accepted to participate in the intervention activities. During three consecutive weeks in August and September 2015, all nurses were invited to a series of seminars. Each seminar lasted 2 hours and covered different topics relating to MMR vaccination i.e measles, MMR vaccination, communication, and autism. The focus was particularly on the epidemiological situation and MMR vaccinations both in the context of Somalia, from where the parents originate, and in the context of Sweden where the parents live currently. In addition, an expert on autism presented the current knowledge of autism and its causes. How to use scientific evidence to debunk the link between autism and MMR vaccination, how to communicate with parents with low vaccine acceptance, and culturally tailor communication were also covered in the seminars. At the end of the third and final seminar, nurses attending the seminar ($n=12$) were asked to participate in a short survey with 11 questions for evaluation purposes. The result of the survey was used to inform the interview guide.

The intervention also included a 14 minute narrative film with Somali role models and experts, entitled "Vaccination – a wise choice for your child”[24]. The film included information about measles and MMR vaccination as well as the experiences of parents, a nurse, and a religious leader. The film was in Somali with Swedish subtitles. The nurses were given a link to the film when it was published on YouTube in 2016 [25]. A two-sided information card was provided to nurses to use in their consultations and to share with parents. The card consisted of key messages in the Somali language on one side with the same information on the other side of the card in Swedish [25]. The key messages focused on; vaccinations offered free of charge for all children, the importance of childhood vaccination to protect against infectious diseases, remember to vaccinate your child before traveling abroad. In addition, a link and QR code to the narrative film and a website with further information were included on the card [25].

Qualitative interviews

Setting and participants

In the fall of 2017, all 12 nurses employed at the CHC in Rinkeby and Tensta were invited to participate in qualitative in-depth interviews. The senior pediatrician at the Regional Preventive Child Health Services facilitated the invitation and recruitment into the study. Three nurses employed after the seminar series held in 2015 were briefed about the seminars and given the materials (film and information card) used in the intervention. Hence, they were deemed eligible for the study and invited to participate in the interviews. Participants were informed in advance about the purpose, design, and methodology of the study. All interviews were conducted in November 2017. In-depth interviews were held with 11 of the nurses who consented. One nurse was on leave during the study period. All nurses participating were female and had a degree in nursing with a specialization in children and youth which is a requirement to work at CHCs as a nurse. The nurses’ working experience at CHCs generally ranged from 3 months to 19 years, with a median of 6 years. Specifically, the nurses had been working at the CHCs in either Rinkeby or Tensta from 3 months up to 17 years, with a median of 4 years, at the time of data collection. Native background of the nurses varied, but was not part of the data collection in order to protect the identity of the nurses in this small sample size.

Data collection

The interviews were semi-structured using a thematic guide (see supplementary file). The outline was adapted from a previous study and also informed by the survey previously conducted after the final seminar [13]. The thematic guide included questions regarding experiences of the interventions, benefits, and changes in daily work since the intervention as well as the perception of parents’ experiences of the interventions. The three nurses who were employed at the CHCs after the seminar series had been held were only asked questions about the parts of the tools in the intervention they had

For peer review only - http://bmjopen.bmj.com/site/about/guidelines.xhtml

been able to use (film and card) and questions about the seminars were excluded. The interview guide was piloted by two members of the research team with minor adaptions made with additional probing questions and reorganizing of a few questions for a better flow. Interviews were conducted in Swedish by the two authors (EA, KGR) and digitally recorded. All interviews were conducted in a private space in the workplace, chosen by the interviewee to allow for openhearted discussions. The interviews ranged from 18 to 43 minutes, 34 minutes on average. Written informed consent was obtained before the start of each interview.

Data analysis
All interviews were transcribed verbatim and analysis was conducted using a thematic analysis [26]. The thematic analysis allowed the analysis to focus on purposely chosen analytic interests regarding the intervention implemented and the tools provided for the nurses. The first author (EA) read the interview transcripts several times to become familiar with the content and thereafter coded by the first author. Two authors (EA, KGR) assessed and discussed initial coding for consistency after which themes were developed. Themes and initial coding were shared among the research team to elaborate and revise the themes until a final version was agreed upon. The analysis was conducted both manually and in Microsoft Office Excel. An example of the analytic process is provided below (table 1) and suitable quotes were selected to support the findings, which are presented in the results.

Table 1. An example of the analytic process.

Text	Code	Subtheme	Overarching theme
But then it probably has with a lot of things to do, maybe with me, that I'm secure in my professional role and also all the work that has been done in various ways	Different things that make me secure in my professional role	Feeling more confident to address parents' MMR vaccine concerns	Perception of improved communication with parents

Patient and public involvement
Patients and the public were not involved in the design of the study.

Ethical considerations
The study received ethical approval for the interviews from the Regional Ethics Committee in Stockholm, Sweden, Dnr 2016/1518-31/5.

Results
An overarching theme emerged, perception of improved communication with parents, and two underlying subthemes were identified;

- Feeling more confident to address parents' MMR vaccine concerns
- Diverse tools as useful support to dispel myth and reduce language barriers.
    - Tailored seminars
    - The information card
    - The film

Perception of improved communication with parents
<u>*Feeling more confident to address parents' MMR vaccine concerns*</u>
The nurses expressed that they felt more confident in engaging in a dialogue with parents discussing MMR vaccination and autism after the intervention. Some nurses described being generally more secure in their professional role whereas other nurses explicitly described being more confident to engage in discussions due to increased knowledge.

For peer review only - http://bmjopen.bmj.com/site/about/guidelines.xhtml

”the more knowledge I have, the more confident I am to engage in discussions” [nurse, #1]

Nurses described that there had been a change in the way they feel regarding discussing MMR vaccination and autism. After the intervention, they felt that they can say with confidence that there is no scientific evidence for the vaccine to cause autism whereas, before the intervention, some nurses expressed feeling insecure and hesitated to vaccinate the child if the parents had low vaccine acceptance. Before the intervention some nurses described that, instead of engaging in a dialogue in such a situation, they just offered the possibility to postpone the MMR vaccination. As two nurses described:

”what if I vaccinate and they get autism?” I was feeling guilty… now I know, it doesn’t cause autism [nurse, #9]

“try to differentiate and say that autism is a separate thing, which I also can explain if you’d like, but the vaccine and the disease are not linked” [nurse, #8]

With reinforced and increased knowledge, nurses felt more secure and confident in saying that there is no scientific evidence for a link to autism, which in turn enabled a better discussion with parents. The dialogue between nurses and parents was facilitated as nurses now felt they could respond to the concerns of parents having low vaccine acceptance and not only describe the MMR vaccine but also confidently communicate the strong scientific evidence of the vaccine not causing autism. When parents brought up that children could become silent due to the vaccine, nurses could name autism and also had the knowledge to explain autism to the parents, elaborating the dialogue and responses to parents.

“feels safe to directly say that there is no evidence” [nurse, #4]

Although the nurses described positive changes, there were still challenges in the dialogue with parents. An issue raised was the delicate balance between discussing proactively versus trying to persuade the parents. In challenging discussions with parents, the nurses did not want to feel like they were a salesperson for the vaccine or persuading the parents in their vaccination decision instead of enabling the parents to make a voluntary decision themselves.

”I don’t want to feel like a salesman when it’s the most challenging” [nurse, #5]

Diverse tools as useful support to dispel myth and reduce language barriers

Tailored seminars

The eight nurses taking part in the seminars were asked questions about their experiences in the interviews. Overall, the nurses expressed that the seminars were beneficial and valuable. The structure and format of the seminars were satisfactory and well received. Moreover, the nurses highlighted that the content and topics included were highly relevant and useful. Particularly, the nurses’ remembered the seminar about the epidemiological situation of measles in Somalia, the Somali vaccination program, and how parents in and from Somalia reason regarding vaccinations as insightful. They also remembered the seminar about autism to be helpful. Gaining cultural insights as well as useful phrases to use in dialogue with parents were particular highlights from the seminar series. Moreover, nurses also emphasized that reinforcing that autism is a disorder with a complex etiology with a hereditary component, has helped them to describe autism to parents having low vaccine acceptance and also debunk the myth regarding the MMR vaccine.

Nurses felt they have been able to take the discussions with parents to another, deeper level after the seminars. They felt more knowledgeable and had practical tools for how to discuss MMR vaccinations and measles in a more structured way with the parents. Some highlighted that learning “one-liners” had been especially useful tools whereas others felt they had more evidence-based information as arguments.

”I received many tools for [use] when I came to work” [nurse, #4]

The nurses expressed that they had increased their knowledge and could dispel the myth of a link between MMR vaccination and autism in their dialogue and discussion with parents. They emphasized that it was very valuable to learn more about the scientific evidence and large epidemiological studies

For peer review only - http://bmjopen.bmj.com/site/about/guidelines.xhtml

that have been conducted regarding MMR vaccinations and autism. Knowing the thorough research and the current solid evidence base made the nurses feel more confident, not only in their own beliefs but also in their conversations and in conveying the information to the parents. The nurses found new ways to approach and motivate the parents and were able to handle the discussion differently when parents had low vaccine acceptance and were worried about autism and MMR vaccination. In addition, nurses felt more knowledgeable regarding autism, its early signs and symptoms, and the complexity of factors potentially involved in the onset of the disease, despite the etiology not yet being clear.

"had the greatest benefit of being able to separate and explain what autism is and what the vaccine is, in my dialogues with parents" [nurse, #6]

"I have found good arguments based on research and [I] can confidently tell it to others, indeed" [nurse, #10]

Information card

Nurses described the usefulness of the card and how it had been helpful to hand it out directly to the parents. They particularly highlighted the link to the film and recommended parents to watch the film at home. Moreover, a stack of cards could lay on the nurse's desk, making it highly visible and easier to remember to hand it out. By handing out the card with information in both Swedish and Somali, the nurses knew exactly the content of the information they gave to the parents directly. In cases of relying on interpreters, some of the nurses were concerned that some of the interpreters may not know how to translate fully the information regarding the diseases and vaccinations correctly and hence the parents may not have received all the information. Thus, by using the card the nurses ensured the parents received the essential and correct information.

"[it has] been really good to hand something over and ask them to watch the film and then come back" [nurse, #9]

The film

Nurses perceived the film positively, particularly since it was a visual tool and available in the Somali language, decreasing language barriers and facilitating a better understanding of the content. The nurses had different ways of using the film. Many nurses recommended parents to watch the film at home with their spouse and also other family members, relatives, or friends, and then they would discuss any questions or concerns raised during the next appointment at CHC. The information card containing the link to the film was often given as a way to promote the film. Other nurses watched the film together with the parents during consultations. Some nurses thought that the film was too long to watch together with parents during appointments as they have a lot to cover during the appointments and not enough time to show a film of 15 minutes. At one of the CHCs, the film was shown on repeat in the waiting room so that parents could watch while waiting for their appointment. On occasions, nurses could sit and watch the film together with parents in the waiting room although some nurses thought it was not an optimal approach as they believed they might single out parents as having low vaccine acceptance.

Nurses emphasized that the film helped parents to reconsider their decision for MMR vaccination and five nurses recalled and gave examples of situations where parents with low vaccine acceptance had changed their mind after watching the movie and chose to vaccinate their child against MMR. Other parents may not have changed their decision regarding MMR vaccination, but the nurses felt that they were less sceptic towards the vaccine.

"came back to vaccinate after having watched the film" [nurse, #1]

"some become softer, the film is really helpful actually" [nurse, #4]

According to the nurses, the parents believed the film was a valuable tool because it included relevant evidence-based information and highlighted the perspective of the parents. Moreover, the film was perceived as credible as most of the experts and parents participating in the film were of Somali origin and spoke Somali. The experts were also well-known, trusted, and respected authorities in the community.

For peer review only - http://bmjopen.bmj.com/site/about/guidelines.xhtml

Nurses expressed that they perceived parents to be less questioning and more open to discussions related to MMR vaccination after the interventions. Parents had become more open to receiving information and having a dialogue. Moreover, the nurses also felt that parents had gained more knowledge, in particular about autism and posed fewer questions about the disease.

"[it is] difficult to measure, it is just a feeling I have" [nurse, #1]

"[I] believe that there are more who are knowledgeable about autism, actually" [nurse, #8]

In addition, the card and the film were especially useful tools during the outbreak of measles after the intervention, as expressed by this nurse:

"During the outbreak we tried to reach out in every way by using information cards, the film, visit to open day care, pediatricians informing…" [nurse, #7]

Discussion

Findings from the present study suggest that tailored interventions with culturally tailored materials can facilitate nurses' communication about vaccinations with parents of Somali descent having low vaccine acceptance. From the perspective of the nurses', the tailored intervention using diverse tools supported the dialogue, dispelled myth and reduced language barriers while also giving nurses cultural insights. Importantly, nurses gained additional confidence in the scientific evidence supporting that MMR vaccination does not cause autism and they were now able to dispel the myth in their dialogue and better convey this information to the parents. The seminars both provided and reinforced knowledge that boosted the nurses' confidence in discussing autism which made them feel more secure when communicating with the parents. Following the intervention and by using the tailored tools provided, the nurses engaged in discussions regarding MMR vaccinations and autism with parents in a different way. Before the intervention, some nurses hesitated themselves and did not address parents when having concerns regarding MMR vaccinations [13]. In addition, tools were also useful during the outbreak of measles in 2017.

Before the intervention was implemented, key barriers were parents fearing that their children would "stop talking" following an MMR vaccination and some parents also described unpleasant encounters with nurses [12, 13]. On the other hand, a key facilitator for vaccinating parents in the community was their trust in nurses [12, 13]. Autism was a sensitive topic that both parents and nurses sidestepped when discussing MMR vaccinations. Similar barriers to vaccination due to fear of autism has also been observed in a Somali community in the US [18, 19].

Factors influencing sub-optimal, vaccination coverage are context specific, complex, and multidimensional [27]. Factors affecting declining vaccine acceptance include barriers in availability and convenience in healthcare systems as well as social determinants, social and cultural norms [28]. In addition, these factors are often specific for their context, setting, type of vaccine and they may also change over time [27]. Limited access to health care and vaccination services for populations has also influenced sub-optimal vaccination coverage in Europe [29].

Using the TIP framework for this intervention project was useful for gaining a thorough understanding of the barriers and drivers for MMR vaccination in the Somali community [30]. Previous studies have highlighted the crucial role of nurses and healthcare providers in parental vaccine acceptance, trust, and decision-making [31-34]. The belief and attitudes of healthcare workers are essential to parental vaccine acceptance and the demand for childhood vaccinations [35, 36]. Hence, it was deemed necessary to tailor interventions for the nurses specifically as part of the multi-component intervention project and to complement the interventions targeting the parents. Multi-component, dialogue-based, and tailored interventions appear to be more effective [37]. In addition, an important aspect of the Swedish national immunization program is the Child Healthcare Services structure and its responsibility for providing and implementing childhood vaccinations [38]. Parents often meet the same nurses for the child's health and development checkups as well as for vaccinations and therefore the nurses have a crucial role in the success of the vaccination program. Although our present study suggests improvements in the dialogue from the perspective of the nurses, the nurses will still be faced

For peer review only - http://bmjopen.bmj.com/site/about/guidelines.xhtml

with recurring challenges and parents having questions and concerns regarding vaccinations. Hence, continued work and effort are needed to support the positive trend in this community. The current study assessed the dialogue and situation 2 years after the implementation of the intervention. Further evaluations of long-term outcomes are needed as well as an assessment of the vaccination coverage in the community over time. As there are workforce turnover, it would be of importance that newly recruited nurses in the community are offered similar training and introduction to the tools. The current intervention was implemented in 2015-2017, repeating the seminars and reinforcing the information for all nurses may also be important for continued capacity building and sustainability. Generally, vaccinating nurses could benefit from training and boosting of knowledge routinely regarding vaccination communication.

Evaluations of tailored interventions are needed to increase the evidence base for effective interventions that can be used by public health authorities to not only increase vaccination acceptance or uptake but also contribute to resilience for national immunization programs. By addressing pockets with suboptimal vaccination coverage and identifying the facilitators and barriers to vaccination, tailored interventions can be designed and implemented to improve vaccination uptake and health equity.

Methodological considerations

Throughout the research process, trustworthiness has been strengthened by several activities. Several members of the interdisciplinary research team had expertise in qualitative methodology and were also familiar with the setting, community, and culture over an extended period before the intervention was implemented. Moreover, the research team familiarized themselves with the data initially and regularly discussed the analysis to strengthen its validity. This study is limited, however, as it only focused on the perspective of the nurses, further studies are needed to capture the perspective of the parents. Although the number of participants in this interview study is small the results are relevant for this specific community and setting, generalizing the findings to other settings should be done with caution.

Conclusion

Nurses' perceived the dialogue to be facilitated and enhanced with parents' vaccination concerns, following the implementation of tailored interventions. Particularly, nurses increased confidence in conveying evidence-based information about autism and MMR vaccination and felt better equipped to convey this information to the parents and to dispel the myth linking autism and MMR in their dialogue with the parents. In addition, the tailored tools were also perceived to reduce language barriers. Child health nurses have a crucial role in the vaccine acceptance and uptake in the Swedish national immunization program and interventions aiming to strengthen the nurses in their communication and dialogue with parents are therefore important. Further studies are needed for assessing the long-term outcomes of the multi-component intervention as well as vaccination coverage within the studied community.

Acknowledgments

The authors wish to thank the Stockholm Region child health services for their support and collaboration in this study. A sincere thank you to all the nurses at the child health care centers who kindly participated with openness and commitment.

Contributors

EA, AJ, AK, AL and KGR designed the study. All authors contributed to the analysis and interpretation of data. EA, AJ and KGR drafted the first version of this paper and AK, AL and AR have revised the paper critically and substantially. The final version of the paper has been approved by all authors and they all agree to be accountable for all aspects of the work.

Funding

The current study was funded by The Public Health Agency of Sweden.

For peer review only - http://bmjopen.bmj.com/site/about/guidelines.xhtml

Conflict of interest

The authors declare no conflict of interest.

Ethical approval

The study received ethical approval for the interviews from the Regional Ethics Committee in Stockholm, Sweden, Dnr 2016/1518-31/5.

Data Sharing Statement

No data are available.

For peer review only

For peer review only - http://bmjopen.bmj.com/site/about/guidelines.xhtml

References

1. European Centre for Disease Prevention and Control. Measles and rubella surveillance – 2017. Stockholm: ECDC; 2018.
2. World Health Organization. Measles emergency in the European Region: WHO; 2019 [Available from: https://www.who.int/europe/emergencies/situations/measles-emergency-in-the-european-region.
3. Folkhälsomyndigheten. Barnvaccinationsprogrammet i Sverige 2020 - Årsrapport. 2021.
4. Folkhälsomyndigheten. Barnvaccinationsprogrammet i Sverige 2019 – Årsrapport. 2020.
5. World Health Organization. SIXTH MEETING OF THE EUROPEAN REGIONAL VERIFICATION COMMISSION FOR MEASLES AND RUBELLA ELIMINATION (RVC). 2017.
6. Folkhälsomyndigheten. Barriers and motivating factors to MMR vaccination in communities with low coverage in Sweden. 2015.
7. Folkhälsomyndigheten. Barnvaccinationsprogrammet i Sverige 2017. 2018.
8. World Health Organization. Tailoring Immunization Programmes (TIP). Copenhagen: WHO Regional Office for Europe; 2019.
9. Butler R, MacDonald NE. Diagnosing the determinants of vaccine hesitancy in specific subgroups: The Guide to Tailoring Immunization Programmes (TIP). Vaccine. 2015.
10. Habersaat KB, Jackson C. Understanding vaccine acceptance and demand—and ways to increase them. Bundesgesundheitsblatt - Gesundheitsforschung - Gesundheitsschutz. 2019.
11. Bystrom E, Lindstrand A, Likhite N, Butler R, Emmelin M. Parental attitudes and decision-making regarding MMR vaccination in an anthroposophic community in Sweden--a qualitative study. Vaccine. 2014;32(50):6752-7.
12. Jama A, Ali M, Lindstrand A, Butler R, Kulane A. Perspectives on the Measles, Mumps and Rubella Vaccination among Somali Mothers in Stockholm. International journal of environmental research and public health. 2018;15(11).
13. Jama A, Lindstrand A, Ali M, Butler R, Kulane A. Nurses' Perceptions Of MMR Vaccine Hesitancy In An Area With Low Vaccination Coverage. Pediatric health, medicine and therapeutics. 2019;10:177-82.
14. Godoy-Ramirez K, Byström E, Lindstrand A, Butler R, Ascher H, Kulane A. Exploring childhood immunization among undocumented migrants in Sweden - following qualitative study and the World Health Organizations Guide to Tailoring Immunization Programmes (TIP). Public health. 2019;171:97-105.
15. Wakefield AJ, Murch SH, Anthony A, Linnell J, Casson DM, Malik M, et al. Ileal-lymphoid-nodular hyperplasia, non-specific colitis, and pervasive developmental disorder in children. Lancet. 1998;351(9103):637-41.
16. Taylor LE, Swerdfeger AL, Eslick GD. Vaccines are not associated with autism: an evidence-based meta-analysis of case-control and cohort studies. Vaccine. 2014;32(29):3623-9.
17. Madsen KM, Hviid A, Vestergaard M, Schendel D, Wohlfahrt J, Thorsen P, et al. A population-based study of measles, mumps, and rubella vaccination and autism. The New England journal of medicine. 2002;347(19):1477-82.
18. Bahta L, Ashkir A. Addressing MMR Vaccine Resistance in Minnesota's Somali Community. Minnesota medicine. 2015;98(10):33-6.
19. Gahr P, DeVries AS, Wallace G, Miller C, Kenyon C, Sweet K, et al. An Outbreak of Measles in an Undervaccinated Community. Pediatrics. 2014;134(1):e220-e8.
20. Paterson P, Meurice F, Stanberry LR, Glismann S, Rosenthal SL, Larson HJ. Vaccine hesitancy and healthcare providers. Vaccine. 2016;34(52):6700-6.
21. Ames HM, Glenton C, Lewin S. Parents' and informal caregivers' views and experiences of communication about routine childhood vaccination: a synthesis of qualitative evidence. Cochrane Database Syst Rev. 2017;2(2):Cd011787.
22. Berry NJ, Danchin M, Trevena L, Witteman HO, Kinnersley P, Snelling T, et al. Sharing knowledge about immunisation (SKAI): An exploration of parents' communication needs to inform development of a clinical communication support intervention. Vaccine. 2018;36(44):6480-90.

For peer review only - http://bmjopen.bmj.com/site/about/guidelines.xhtml

23. Karafillakis E, Dinca I, Apfel F, Cecconi S, Wűrz A, Takacs J, et al. Vaccine hesitancy among healthcare workers in Europe: A qualitative study. Vaccine. 2016;34(41):5013-20.
24. Jama A, Appelqvist E, Kulane A, Karregård S, Rubin J, Nejat S, et al. Design and implementation of tailored intervention to increase vaccine acceptance in a Somali community in Stockholm, Sweden - based on the Tailoring Immunization Programmes approach. Public Health in Practice. 2022;4:100305.
25. Folkhälsomyndigheten. Informationsmaterial om vaccinationer på somaliska 2022 [Available from: https://www.folkhalsomyndigheten.se/smittskydd-beredskap/vaccinationer/informationsmaterial-om-vaccinationer/informationsmaterial-om-vaccinationer-pa-somaliska/.
26. Braun V, Clarke V. Using thematic analysis in psychology. Qualitative Research in Psychology. 2006;3(2):77-101.
27. MacDonald NE. Vaccine hesitancy: Definition, scope and determinants. Vaccine. 2015;33(34):4161-4.
28. Dubé E, Gagnon D, MacDonald N, Bocquier A, Peretti-Watel P, Verger P. Underlying factors impacting vaccine hesitancy in high income countries: a review of qualitative studies. Expert Review of Vaccines. 2018;17(11):989-1004.
29. Boyce T, Gudorf A, de Kat C, Muscat M, Butler R, Habersaat KB. Towards equity in immunisation. Euro Surveill. 2019;24(2).
30. Jama A, Appelqvist E, Kulane A, Karregård S, Rubin J, Nejat S, et al. Design and implementation of tailored intervention to increase vaccine acceptance in a Somali community in Stockholm, Sweden - based on the Tailoring Immunizations Programs approach. Submitted. 2022.
31. Leask J, Kinnersley P, Jackson C, Cheater F, Bedford H, Rowles G. Communicating with parents about vaccination: a framework for health professionals. BMC pediatrics. 2012;12:154.
32. Gust DA, Darling N, Kennedy A, Schwartz B. Parents with doubts about vaccines: which vaccines and reasons why. Pediatrics. 2008;122(4):718-25.
33. Glanz JM, Wagner NM, Narwaney KJ, Shoup JA, McClure DL, McCormick EV, et al. A mixed methods study of parental vaccine decision making and parent-provider trust. Academic pediatrics. 2013;13(5):481-8.
34. Cooper LZ, Larson HJ, Katz SL. Protecting public trust in immunization. Pediatrics. 2008;122(1):149-53.
35. Simone B, Carrillo-Santisteve P, Lopalco PL. Healthcare workers role in keeping MMR vaccination uptake high in Europe: a review of evidence. Euro surveillance : bulletin europeen sur les maladies transmissibles = European communicable disease bulletin. 2012;17(26):20206.
36. Dube E. Addressing vaccine hesitancy: the crucial role of healthcare providers. Clin Microbiol Infect. 2017;23(5):279-80.
37. Dubé E, Gagnon D, MacDonald NE. Strategies intended to address vaccine hesitancy: Review of published reviews. Vaccine. 2015;33(34):4191-203.
38. Wettergren B, Blennow M, Hjern A, Söder O, Ludvigsson JF. Child Health Systems in Sweden. The Journal of Pediatrics. 2016;177:S187-S202.

For peer review only

For peer review only - http://bmjopen.bmj.com/site/about/guidelines.xhtml

Folkhälsomyndigheten
PUBLIC HEALTH AGENCY OF SWEDEN

TIP Sweden: Evaluation of tailored communication on vaccination in Rinkeby and Tensta, Stockholm

Interview Guide – Nurses at the Child Health Clinics

Aim: To investigate how the implemented interventions are experienced and received by CHC nurses and how the interventions have influenced their work towards vaccine-hesitant parents.

The interview

- Give a brief introduction on the purpose of the interview and procedures, as a follow-up to previous written information
- Consent to participate in the interview, recording, etc.

Date (yy/mm/dd):____/____/____

Code no. for the interview:

Place for the interview: ____________

Starting time: __:____ End: ___:____

Interviever: ____

Background information

- Employment title
- How long have you worked within the CHC/services?
- District you are working in (Rinkeby or Tensta)?
- How long have you worked in the district?

Your experience about the tailored interventions organized by the PHAS

- A series of three seminars was given during autumn in 2015, did you participate?
- Can you describe what you thought about the content of the seminars? What was good/less good?
- Did the seminars give you new knowledge? (About autism, cultural communication, summary about Wakefields research, diseases and the global situation?)
- Can you describe the discussions that followed the seminars?
- Have you been able to use the information from the lectures when you meet vaccine-hesitant parents? (Can you give an example of a recent conversation with a VH parent? what happened, what did they say, what was the outcome etc. How did they use the training? Perhaps ask for an example where the conversation went well, and one where it did not. ?)
- Would you like the seminars to be given again? (In what format? Themes?)
- Can you describe other interventions/competence enhancement activities related to vaccine hesitancy organized within the CHS? (What do you think about these? Have they been in useful, how?)

For peer review only - http://bmjopen.bmj.com/site/about/guidelines.xhtml

Benefits from interventions by PHAS in your daily work

- Are you familiar with the other intervention activities within the project, targeting the Somali speaking parents? (What do you think about them? Somali film, seminars, peers, postcard)
- Have you had any use of the interventions, generally? Can you describe if / how you have benefited from these in your work? (Referred to the parents? Used for discussion))
- Do you know about the peer group created within the project? (Have you met anyone in the group? Collaborated in any way? Have they served as support in your work)

Changed mode of work following the intervention

- Has your mode of work changed or been affected since the time before the interventions started? (In what way? Regarding autism?)
- Do you as nurses work differently at the CHC when approaching vaccine-hesitant parents? (Can you describe how? Internal communication? Autism?)
- Can you identify other / new groups of parents in the neighborhood who are hesitant towards vaccinations?

Your perception on parents' experiences of the interventions and change in parents' knowledge

- How do you think that Somali speaking parents experience the interventions? (Have they been reached? Their attitude towards the activities, seminars, films, peers)
- What questions and concerns do the parents have today? (How do they reason?)
- Do you experience any differences in VH parent´s knowledge compared with the time before the intervention?
- Do you experience any differences in attitudes among vaccine-hesitant parents compared with the time before the intervention? (What differences? What about their perception of social norms around vaccination in the community? (social opportunity COM-B) Larger or minor problem?)
- How do parents reason about autism today? (Any changes? How?)
- Do you experience a difference in behavior (decision to vaccinate) among vaccine-hesitant parents as compared to how it was before the interventions? Do they perceive a difference in decision-making in parents (reflective versus automatic motivation COM-B)

Future perspectives

- Do you need any further support in specific questions/areas regarding vaccination? (Which areas? Format? Something you have missed? Other interventions?)
- Do you need further knowledge on specific issues related to vaccine-hesitancy? (Which? How would you like to have knowledge?)
- Do you have any suggestion on other ways to reach vaccine-hesitant parents (which the staff at the CHC can work with?
- Do you have any thoughts about the group of peers? (Any benefit from their work, suggestions for cooperation, joint activities)?

Before we finish the interview

- Is there anything else you would like to address or add that we have not yet discussed?

Thank you for your participation!

Solna Folkhälsomyndigheten, SE-171 82 Solna. Visit: Nobels väg 18. **Östersund** Folkhälsomyndigheten, SE-831 40 Östersund. Visit: Forskarens väg 3.
Telephone + 46 10 205 20 00 **E-mail** info@folkhalsomyndigheten.se **www.folkhalsomyndigheten.se**

For peer review only - http://bmjopen.bmj.com/site/about/guidelines.xhtml

Standards for Reporting Qualitative Research (SRQR)*

http://www.equator-network.org/reporting-guidelines/srqr/

Title and abstract

	Page/line no(s).
**Title** - Concise description of the nature and topic of the study Identifying the study as qualitative or indicating the approach (e.g., ethnography, grounded theory) or data collection methods (e.g., interview, focus group) is recommended	Page 1
**Abstract** - Summary of key elements of the study using the abstract format of the intended publication; typically includes background, purpose, methods, results, and conclusions	Page 1

Introduction

**Problem formulation** - Description and significance of the problem/phenomenon studied; review of relevant theory and empirical work; problem statement	Introduction, page 3-4
**Purpose or research question** - Purpose of the study and specific objectives or questions	End of introduction, page 4

Methods

**Qualitative approach and research paradigm** - Qualitative approach (e.g., ethnography, grounded theory, case study, phenomenology, narrative research) and guiding theory if appropriate; identifying the research paradigm (e.g., postpositivist, constructivist/ interpretivist) is also recommended; rationale**	Methods, page 5-6
**Researcher characteristics and reflexivity** - Researchers' characteristics that may influence the research, including personal attributes, qualifications/experience, relationship with participants, assumptions, and/or presuppositions; potential or actual interaction between researchers' characteristics and the research questions, approach, methods, results, and/or transferability	Methods & methodological considerations, page 5-6 & 10
**Context** - Setting/site and salient contextual factors; rationale**	Methods, page 5-6
**Sampling strategy** - How and why research participants, documents, or events were selected; criteria for deciding when no further sampling was necessary (e.g., sampling saturation); rationale**	Methods, page 5-6
**Ethical issues pertaining to human subjects** - Documentation of approval by an appropriate ethics review board and participant consent, or explanation for lack thereof; other confidentiality and data security issues	Methods, page 6
**Data collection methods** - Types of data collected; details of data collection procedures including (as appropriate) start and stop dates of data collection and analysis, iterative process, triangulation of sources/methods, and modification of procedures in response to evolving study findings; rationale**	Methods, page 5-6
**Data collection instruments and technologies** - Description of instruments (e.g., interview guides, questionnaires) and devices (e.g., audio recorders) used for data collection; if/how the instrument(s) changed over the course of the study	Methods, page 5-6 and also interview guide in appendix
**Units of study** - Number and relevant characteristics of participants, documents, or events included in the study; level of participation (could be reported in results)	Methods, page 5-6

For peer review only - http://bmjopen.bmj.com/site/about/guidelines.xhtml

For peer review only

**Data processing** - Methods for processing data prior to and during analysis, including transcription, data entry, data management and security, verification of data integrity, data coding, and anonymization/de-identification of excerpts	Methods, page 5-6
**Data analysis** - Process by which inferences, themes, etc., were identified and developed, including the researchers involved in data analysis; usually references a specific paradigm or approach; rationale**	Methods, page 5-6
**Techniques to enhance trustworthiness** - Techniques to enhance trustworthiness and credibility of data analysis (e.g., member checking, audit trail, triangulation); rationale**	Methodological considerations, page 10

Results/findings

**Synthesis and interpretation** - Main findings (e.g., interpretations, inferences, and themes); might include development of a theory or model, or integration with prior research or theory	Results, pages 6-9
**Links to empirical data** - Evidence (e.g., quotes, field notes, text excerpts, photographs) to substantiate analytic findings	Quotes included throughout the in results section, pages 6-9

Discussion

**Integration with prior work, implications, transferability, and contribution(s) to the field -** Short summary of main findings; explanation of how findings and conclusions connect to, support, elaborate on, or challenge conclusions of earlier scholarship; discussion of scope of application/generalizability; identification of unique contribution(s) to scholarship in a discipline or field	Pages 9-10
**Limitations** - Trustworthiness and limitations of findings	Methodological considerations, page 10

Other

**Conflicts of interest** - Potential sources of influence or perceived influence on study conduct and conclusions; how these were managed	See statement, page 11
**Funding** - Sources of funding and other support; role of funders in data collection, interpretation, and reporting	See statement, page 10

*The authors created the SRQR by searching the literature to identify guidelines, reporting standards, and critical appraisal criteria for qualitative research; reviewing the reference lists of retrieved sources; and contacting experts to gain feedback. The SRQR aims to improve the transparency of all aspects of qualitative research by providing clear standards for reporting qualitative research.
**The rationale should briefly discuss the justification for choosing that theory, approach, method, or technique rather than other options available, the assumptions and limitations implicit in those choices, and how those choices influence study conclusions and transferability. As appropriate, the rationale for several items might be discussed together.

Reference:

O'Brien BC, Harris IB, Beckman TJ, Reed DA, Cook DA. **Standards for reporting qualitative research: a synthesis of recommendations.** *Academic Medicine*, Vol. 89, No. 9 / Sept 2014
DOI: 10.1097/ACM.0000000000000388

For peer review only - http://bmjopen.bmj.com/site/about/guidelines.xhtml

BMJ Open

Exploring nurses' experiences of a tailored intervention to increase MMR vaccine acceptance in a Somali community in Stockholm, Sweden: a qualitative interview study

Journal:	*BMJ Open*
Manuscript ID	bmjopen-2022-067169.R2
Article Type:	Original research
Date Submitted by the Author:	19-Jan-2023
Complete List of Authors:	Appelqvist, Emma; Public Health Agency of Sweden, Department of Public Health Analysis and Data Management; Lund University, Clinical Microbiology, Department of Translational Medicine<br>Jama, Asha; Public Health Agency of Sweden, Department of Public Health Analysis and Data Management; Karolinska Institutet, Department of Global Public Health<br>Kulane, Asli; Karolinska Institutet, Department of Global Public Health<br>Roth, Adam; Lund University, Clinical Microbiology, Department of Translational Medicine; Public Health Agency of Sweden, Department of Public Health Analysis and Data Management<br>Lindstrand, Ann; World Health Organization, Department of Immunization, Vaccines and Biologicals, Unit Essential Programme on Immunization<br>Godoy-Ramirez, Karina; Public Health Agency of Sweden, The Office of the Head for Communicable Disease Control and Health Protection
**Primary Subject Heading**:	Public health
Secondary Subject Heading:	Qualitative research
Keywords:	Public health < INFECTIOUS DISEASES, Paediatric infectious disease & immunisation < PAEDIATRICS, PUBLIC HEALTH

SCHOLARONE™
Manuscripts

BMJ

I, the Submitting Author has the right to grant and does grant on behalf of all authors of the Work (as defined in the below author licence), an exclusive licence and/or a non-exclusive licence for contributions from authors who are: i) UK Crown employees; ii) where BMJ has agreed a CC-BY licence shall apply, and/or iii) in accordance with the terms applicable for US Federal Government officers or employees acting as part of their official duties; on a worldwide, perpetual, irrevocable, royalty-free basis to BMJ Publishing Group Ltd ("BMJ") its licensees and where the relevant Journal is co-owned by BMJ to the co-owners of the Journal, to publish the Work in this journal and any other BMJ products and to exploit all rights, as set out in our licence.

The Submitting Author accepts and understands that any supply made under these terms is made by BMJ to the Submitting Author unless you are acting as an employee on behalf of your employer or a postgraduate student of an affiliated institution which is paying any applicable article publishing charge ("APC") for Open Access articles. Where the Submitting Author wishes to make the Work available on an Open Access basis (and intends to pay the relevant APC), the terms of reuse of such Open Access shall be governed by a Creative Commons licence – details of these licences and which Creative Commons licence will apply to this Work are set out in our licence referred to above.

Other than as permitted in any relevant BMJ Author's Self Archiving Policies, I confirm this Work has not been accepted for publication elsewhere, is not being considered for publication elsewhere and does not duplicate material already published. I confirm all authors consent to publication of this Work and authorise the granting of this licence.

For peer review only

Exploring nurses' experiences of a tailored intervention to increase MMR vaccine acceptance in a Somali community in Stockholm, Sweden: a qualitative interview study

Emma Appelqvist, Asha Jama, Asli Kulane, Adam Roth, Ann Lindstrand och Karina Godoy-Ramirez

Emma Appelqvist, MPH, PhD student
Affiliation: Public Health Agency of Sweden, Stockholm, Sweden
Department of Public Health Analysis and Data Management
Address: Nobels väg 18, SE-171 82 Solna, Sweden
Tel.: +46 (0)10 205 2022, Email: Emma.Appelqvist@folkhalsomyndigheten.se

Affiliation: Lund University, Lund, Sweden
Faculty of Medicine, Clinical Microbiology, Department of Translational Medicine
Address: Jan Waldenströms gata 59, 205 02 Malmö, Sweden

Asha Jama, MPH
Affiliation: Public Health Agency of Sweden, Stockholm, Sweden
Department of Public Health Analysis and Data Management
Address: Nobels väg 18, SE-171 82 Solna, Sweden
Tel.: +46 (0)10 205 2082, Email: Asha.Jama@folkhalsomyndigheten.se

Affiliation: Karolinska Institutet, Stockholm, Sweden
Department of Global Public Health
Address: Tomtebodavägen 18A, SE-171 77 Stockholm, Sweden

Asli Kulane, MD, MSc, PhD, Associate Professor
Affiliation: Karolinska Institutet, Stockholm, Sweden
Department of Global Public Health
Address: Tomtebodavägen 18A, SE-171 77 Stockholm, Sweden
Tel.: +46 (0) 8-524 833 88, Email: Asli.Kulane@ki.se

Adam Roth, MD, PhD, Associate Professor
Affiliation: Public Health Agency of Sweden, Stockholm, Sweden
Department of Public Health Analysis and Data Management
Address: Nobels väg 18, SE-171 82 Solna, Sweden
Email; adam.roth@med.lu.se

Affiliation: Lund University, Lund, Sweden
Faculty of Medicine, Clinical Microbiology, Department of Translational Medicine
Address: Jan Waldenströms gata 59, 205 02 Malmö, Sweden

Ann Lindstrand, MD, MPH, PhD
Affiliation: World Health Organization (WHO) Headquarters, Geneva, Switzerland
Department of Immunization, Vaccines and Biologicals, Unit Essential Programme on Immunization
Address: 20 Avenue Appia, 1211 Geneva 27, Switzerland
Tel.: +41 227913436, Email: lindstranda@who.int

Karina Godoy-Ramirez, MSc, MPH, PhD
Affiliation: Public Health Agency of Sweden, Stockholm, Sweden
The Office of the Head for Communicable Disease Control and Health Protection
Address: Nobels väg 18, SE-171 82 Solna, Sweden
Tel.: +46 (0)10 205 2668, Email: Karina.Godoy@folkhalsomyndigheten.se

For peer review only - http://bmjopen.bmj.com/site/about/guidelines.xhtml

Correspondence to:
Emma Appelqvist
Emma.Appelqvist@folkhalsomyndigheten.se

Abstract

Objectives

To explore nurses' experiences of a tailored intervention that supported them with knowledge and tools to use during encounters and dialogue with parents with low vaccine acceptance.

Design

A qualitative study with in-depth interviews conducted in 2017. Data were analyzed using thematic analysis.

Setting

This study was part of a multi-component intervention targeting Somali parents and the nurses at child health centers in the Rinkeby and Tensta neighborhoods of Stockholm. An area with documented low MMR vaccination coverage. Previous research has revealed that Somali parents in the community delayed MMR vaccination due to fear of autism despite lack of scientific evidence. The interventions were implemented in 2015-2017.

Participants

Eleven nurses employed at the child health centers involved in the intervention participated in interviews. The tailored intervention targeting nurses included a series of seminars, a narrative film, and an information card with key messages for distribution to parents.

Results

The qualitative analysis revealed an overarching theme: perception of improved communication with parents. Two underlying themes were identified (1) feeling more confident to address parents' MMR vaccine concerns (2) diverse tools as useful support to dispel myth and reduce language barriers.

Conclusion

From the nurses' perspective, the tailored intervention was useful to improve communication with parents having vaccine concerns. Nurses have a crucial role in vaccine uptake and acceptance. Interventions aiming to strengthen their communication with parents are therefore essential, especially in areas with lower vaccine acceptance.

For peer review only

Strengths and limitations of this study

- Qualitative interviews and thematic analysis enabled a deeper understanding of nurses' experiences of a tailored intervention supporting encounters and dialogue with parents having low vaccine acceptance.
- In-depth interviews of nurses provided unique insights into their experiences of using tailored tools.
- This study is limited as it only focused on the perspective of the nurses; further studies are needed to capture the perspective of the parents.
- The results are relevant for the specific community and setting for the study; caution should be used in attempting to generalize the findings to other settings.

Introduction

Cases of measles increased in Europe and the WHO European Region in 2017-2019, although the disease could be prevented and even eradicated by effective vaccines [1, 2]. In the WHO European Region more than 82 000 cases were reported [2]. The majority of the cases occurred among under-immunized individuals and in pockets of lower vaccination coverage below the recommended threshold of 95% coverage for achieving herd immunity [1, 2].

The WHO Regional Office for Europe developed an approach, the Tailoring Immunization Programs (TIP), aiming to facilitate a deeper understanding of barriers and drivers for suboptimal vaccination coverage in particular communities [3]. Based on findings of barriers and drivers, tailored and targeted interventions may be designed and implemented to support improved and equitable vaccination uptake [4]. The TIP approach is based on scientific evidence from social and behavioral science research, underpinned by the Capability-Opportunity-Motivation-Behaviour (COM-B) model and the Behaviour Change Wheel (BCW) framework which has been adapted for behavioral insights for vaccinations [3, 5].

In Sweden, the measles, mumps, rubella (MMR) vaccination coverage has been high and stable throughout the last decade, with 97.1 % of two-year children vaccinated against MMR in 2020 [6]. The national immunization program (NIP) in Sweden is voluntary and all vaccinations are offered free of charge. The first dose of MMR vaccination is offered at 18 months at child health care centers (CHC) and a second dose is offered by school health services in grades 1 and 2 (children aged 7 to 8 years). Cases of measles in Sweden have varied from 3 to 51 cases per year during the last ten years [7]. All recent cases have either been imported or epidemiologically linked to an imported case, apart from a few sporadic cases of unknown origin [personal communication, Hélène Englund, The Public Health Agency of Sweden, 2022-11-15]. The Regional verification committee of WHO concluded measles and rubella to be eliminated in Sweden in 2015 and 2017, respectively [8]. There are, however, regional and local pockets in Sweden with lower MMR vaccination coverage such as. Rinkeby and Tensta districts in the Region of Stockholm where there is a high percentage of residents with foreign backgrounds, approximately 30% of these are of Somali origin [9]. In 2000 the vaccination coverage for MMR for two-year-olds in Rinkeby and Tensta started to decline, and between 2002 and 2013, the vaccination coverage stabilized at around 70 %. With the accumulation of susceptible unimmunized populations, the Rinkeby and Tensta area had an increased risk of measles outbreaks. Between January to March spring 2017, there was a measles outbreak in the area, which afflicted the Somali community. During the outbreak 12 cases of measles were reported, of which nine cases were children [10]. In 2013, the Public Health Agency of Sweden (PHAS) piloted the TIP approach and conducted formative research (TIP phase 1-2, based on the TIP Guide version 1, 2013) in three communities with known or suspected suboptimal MMR vaccination coverage; anthroposophic, Somali, and undocumented migrants [11-14].

Results from previous qualitative studies among parents in the Somali community in this area showed that parents had low vaccine acceptance and delayed MMR vaccination primarily due to fear that children would “stop talking“ and develop autism [12]. Concerns about vaccine-induced autism due to MMR vaccination originate from the spurious, retracted article published by Wakefield et al. in 1998 which suggested a link between the MMR vaccine and autism [15]. No evidence for such a link between autism and MMR vaccines has been found in several extensive international studies [16, 17]. Despite the lack of evidence, the myth has been rooted among Somali parents in Rinkeby and Tensta for years [12]. Similarly, lower vaccination coverage for MMR has been observed in Somali communities in the US, again due to fear of autism and possible links to the vaccine [18, 19]. Previous studies have shown that parents in Rinkeby and Tensta would attend all appointments at the CHC but refrain from attending the 18 months appointment at which the MMR vaccine is offered. Thus, access and convenience for vaccination services were not deemed to be a barrier for parents with low vaccine acceptance as parents attended their CHC. Moreover, it became evident that nurses at the CHC did not address parents’ concerns regarding MMR vaccination [13] Previous research has highlighted the crucial role of nurses in parents’ decision-making about vaccinations [20-23]. As, having an open and accepting approach is key in discussions regarding vaccinations [21, 22], multi-component tailored

For peer review only - http://bmjopen.bmj.com/site/about/guidelines.xhtml

interventions were therefore designed and implemented in 2015-2017 for two main target groups in Rinkeby and Tensta; Somali parents and CHC nurses. The design and implementation of these interventions have been described in detail previously [24]. Although the intervention project had two main target groups, this study focuses only on the evaluation of those intervention activities for nurses. This current study aimed to explore nurses' experiences of a tailored intervention that supported them with knowledge and tools to use during encounters and dialogue with parents with low vaccine acceptance.

Methods

Intervention activities and tools

The intervention activities for nurses included education and training as well as a narrative film with Somali role models and information card to use as tools when interacting with parents. The overall design and implementation of the intervention for both parents and nurses have been described in previously published research [24]. All 12 nurses working at the CHCs in either Rinkeby or Tensta at the time of implementation were offered and accepted to participate in the intervention activities. During three consecutive weeks in August and September 2015, all nurses were invited to a series of seminars. Each seminar lasted 2 hours and covered different topics relating to MMR vaccination i.e measles, MMR vaccination, communication, and autism. The focus was particularly on the epidemiological situation and MMR vaccinations both in the context of Somalia, from where the parents originate, and in the context of Sweden where the parents live currently. In addition, an expert on autism presented the current knowledge of autism and its causes. How to use scientific evidence to debunk the link between autism and MMR vaccination, how to communicate with parents with low vaccine acceptance, and culturally tailor communication were also covered in the seminars. At the end of the third and final seminar, nurses attending the seminar ($n=12$) were asked to participate in a short survey with 11 questions for evaluation purposes. The result of the survey was used to inform the interview guide.

The intervention also included a 14 minute narrative film with Somali role models and experts, entitled "Vaccination – a wise choice for your child"[24]. The film included information about measles and MMR vaccination as well as the experiences of parents, a nurse, and a religious leader. The film was in Somali with Swedish subtitles. The nurses were given a link to the film when it was published on YouTube in 2016 [25]. A two-sided information card was provided to nurses to use in their consultations and to share with parents. The card consisted of key messages in the Somali language on one side with the same information on the other side of the card in Swedish [25]. The key messages focused on; vaccinations offered free of charge for all children, the importance of childhood vaccination to protect against infectious diseases, remember to vaccinate your child before traveling abroad. In addition, a link and QR code to the narrative film and a website with further information were included on the card [25].

Qualitative interviews

Setting and participants

In the fall of 2017, all 12 nurses employed at the CHC in Rinkeby and Tensta were invited to participate in qualitative in-depth interviews. The senior pediatrician at the Regional Preventive Child Health Services facilitated the invitation and recruitment into the study. Three nurses employed after the seminar series held in 2015 were briefed about the seminars and given the materials (film and information card) used in the intervention. Hence, they were deemed eligible for the study and invited to participate in the interviews. Participants were informed in advance about the purpose, design, and methodology of the study. All interviews were conducted in November 2017. In-depth interviews were held with 11 of the nurses who consented. One nurse was on leave during the study period. All nurses participating were female and had a degree in nursing with a specialization in children and youth which is a requirement to work at CHCs as a nurse. The nurses' working experience at CHCs generally ranged from 3 months to 19 years, with a median of 6 years. Specifically, the nurses had been working at the CHCs in either Rinkeby or Tensta from 3 months up to 17 years, with a median of

For peer review only - http://bmjopen.bmj.com/site/about/guidelines.xhtml

4 years, at the time of data collection. Native background of the nurses varied, but was not part of the data collection in order to protect the identity of the nurses in this small sample size.

Data collection

The interviews were semi-structured using a thematic guide (see supplementary file). The outline was adapted from a previous study and also informed by the survey previously conducted after the final seminar [13]. The thematic guide included questions regarding experiences of the interventions, benefits, and changes in daily work since the intervention as well as the perception of parents' experiences of the interventions. The three nurses who were employed at the CHCs after the seminar series had been held were only asked questions about the parts of the tools in the intervention they had been able to use (film and card) and questions about the seminars were excluded. The interview guide was piloted by two members of the research team with minor adaptions made with additional probing questions and reorganizing of a few questions for a better flow. Interviews were conducted in Swedish by the two authors (EA, KGR) and digitally recorded. All interviews were conducted in a private space in the workplace, chosen by the interviewee to allow for open discussions. The interviews ranged from 18 to 43 minutes, 34 minutes on average. Written informed consent was obtained before the start of each interview.

Data analysis

All interviews were transcribed verbatim and analysis was conducted using a thematic analysis [26]. The thematic analysis allowed the analysis to focus on purposely chosen analytic interests regarding the intervention implemented and the tools provided for the nurses. The first author (EA) read the interview transcripts several times to become familiar with the content and thereafter coded by the first author. Two authors (EA, KGR) assessed and discussed initial coding for consistency after which themes were developed. Themes and initial coding were shared among the research team to elaborate and revise the themes until a final version was agreed upon. The analysis was conducted both manually and in Microsoft Office Excel. An example of the analytic process is provided below (table 1) and suitable quotes were selected to support the findings, which are presented in the results.

Table 1. An example of the analytic process

Text	Code	Subtheme	Overarching theme
But then it probably has with a lot of things to do, maybe with me, that I'm secure in my professional role and also all the work that has been done in various ways	Different things that make me secure in my professional role	Feeling more confident to address parents' MMR vaccine concerns	Perception of improved communication with parents

Ethical considerations

The study received ethical approval for the interviews from the Regional Ethics Committee in Stockholm, Sweden, Dnr 2016/1518-31/5.

Patient and public involvement

None.

Results

An overarching theme emerged, perception of improved communication with parents, and two underlying subthemes were identified:

- Feeling more confident to address parents' MMR vaccine concerns
- Diverse tools as useful support to dispel myth and reduce language barriers.

For peer review only - http://bmjopen.bmj.com/site/about/guidelines.xhtml

- Tailored seminars
- The information card
- The film

Perception of improved communication with parents

Feeling more confident to address parents' MMR vaccine concerns

The nurses expressed that they felt more confident in engaging in a dialogue with parents discussing MMR vaccination and autism after the intervention. Some nurses described being generally more secure in their professional role whereas other nurses explicitly described being more confident to engage in discussions due to increased knowledge.

"the more knowledge I have, the more confident I am to engage in discussions" [nurse, #1]

Nurses described that there had been a change in the way they feel regarding discussing MMR vaccination and autism. After the intervention, they felt that they can say with confidence that there is no scientific evidence for the vaccine to cause autism whereas, before the intervention, some nurses expressed feeling insecure and hesitated to vaccinate the child if the parents had low vaccine acceptance. Before the intervention some nurses described that, instead of engaging in a dialogue in such a situation, they just offered the possibility to postpone the MMR vaccination. As two nurses described:

"what if I vaccinate and they get autism?" I was feeling guilty… now I know, it doesn't cause autism [nurse, #9]

"try to differentiate and say that autism is a separate thing, which I also can explain if you'd like, but the vaccine and the disease are not linked" [nurse, #8]

With reinforced and increased knowledge, nurses felt more secure and confident in saying that there is no scientific evidence for a link to autism, which in turn enabled a better discussion with parents. The dialogue between nurses and parents was facilitated as nurses now felt they could respond to the concerns of parents having low vaccine acceptance and not only describe the MMR vaccine but also confidently communicate the strong scientific evidence of the vaccine not causing autism. When parents brought up that children could become silent due to the vaccine, nurses could name autism and also had the knowledge to explain autism to the parents, elaborating the dialogue and responses to parents.

"feels safe to directly say that there is no evidence" [nurse, #4]

Although the nurses described positive changes, there were still challenges in the dialogue with parents. An issue raised was the delicate balance between discussing proactively versus trying to persuade the parents. In challenging discussions with parents, the nurses did not want to feel like they were a salesperson for the vaccine or persuading the parents in their vaccination decision instead of enabling the parents to make a voluntary decision themselves.

"I don't want to feel like a salesman when it's the most challenging" [nurse, #5]

Diverse tools as useful support to dispel myth and reduce language barriers

<u>Tailored seminars</u>

The eight nurses taking part in the seminars were asked questions about their experiences in the interviews. Overall, the nurses expressed that the seminars were beneficial and valuable. The structure and format of the seminars were satisfactory and well received. Moreover, the nurses highlighted that the content and topics included were highly relevant and useful. Particularly, the nurses' remembered the seminar about the epidemiological situation of measles in Somalia, the Somali vaccination program, and how parents in and from Somalia reason regarding vaccinations as insightful. They also remembered the seminar about autism to be helpful. Gaining cultural insights as well as useful phrases to use in dialogue with parents were particular highlights from the seminar series. Moreover, nurses also emphasized that reinforcing that autism is a disorder with a complex etiology with a hereditary component, has helped them to describe autism to parents having low vaccine acceptance and also debunk the myth regarding the MMR vaccine.

For peer review only - http://bmjopen.bmj.com/site/about/guidelines.xhtml

Nurses felt they have been able to take the discussions with parents to another, deeper level after the seminars. They felt more knowledgeable and had practical tools for how to discuss MMR vaccinations and measles in a more structured way with the parents. Some highlighted that learning “one-liners” had been especially useful tools whereas others felt they had more evidence-based information as arguments.

”I received many tools for [use] when I came to work” [nurse, #4]

The nurses expressed that they had increased their knowledge and could dispel the myth of a link between MMR vaccination and autism in their dialogue and discussion with parents. They emphasized that it was very valuable to learn more about the scientific evidence and large epidemiological studies that have been conducted regarding MMR vaccinations and autism. Knowing the thorough research and the current solid evidence base made the nurses feel more confident, not only in their own beliefs but also in their conversations and in conveying the information to the parents. The nurses found new ways to approach and motivate the parents and were able to handle the discussion differently when parents had low vaccine acceptance and were worried about autism and MMR vaccination. In addition, nurses felt more knowledgeable regarding autism, its early signs and symptoms, and the complexity of factors potentially involved in the onset of the disease, despite the etiology not yet being clear.

“had the greatest benefit of being able to separate and explain what autism is and what the vaccine is, in my dialogues with parents” [nurse, #6]

“I have found good arguments based on research and [I] can confidently tell it to others, indeed” [nurse, #10]

<u>Information card</u>

Nurses described the usefulness of the card and how it had been helpful to hand it out directly to the parents. They particularly highlighted the link to the film and recommended parents to watch the film at home. Moreover, a stack of cards could lay on the nurse’s desk, making it highly visible and easier to remember to hand it out. By handing out the card with information in both Swedish and Somali, the nurses knew exactly the content of the information they gave to the parents directly. In cases of relying on interpreters, some of the nurses were concerned that some of the interpreters may not know how to translate fully the information regarding the diseases and vaccinations correctly and hence the parents may not have received all the information. Thus, by using the card the nurses ensured the parents received the essential and correct information.

”[it has] been really good to hand something over and ask them to watch the film and then come back” [nurse, #9]

<u>The film</u>

Nurses perceived the film positively, particularly since it was a visual tool and available in the Somali language, decreasing language barriers and facilitating a better understanding of the content. The nurses had different ways of using the film. Many nurses recommended parents to watch the film at home with their spouse and also other family members, relatives, or friends, and then they would discuss any questions or concerns raised during the next appointment at CHC. The information card containing the link to the film was often given as a way to promote the film. Other nurses watched the film together with the parents during consultations. Some nurses thought that the film was too long to watch together with parents during appointments as they have a lot to cover during the appointments and not enough time to show a film of 15 minutes. At one of the CHCs, the film was shown on repeat in the waiting room so that parents could watch while waiting for their appointment. On occasions, nurses could sit and watch the film together with parents in the waiting room although some nurses thought it was not an optimal approach as they believed they might single out parents as having low vaccine acceptance.

Nurses emphasized that the film helped parents to reconsider their decision for MMR vaccination and five nurses recalled and gave examples of situations where parents with low vaccine acceptance had changed their mind after watching the movie and chose to vaccinate their child against MMR. Other

For peer review only - http://bmjopen.bmj.com/site/about/guidelines.xhtml

parents may not have changed their decision regarding MMR vaccination, but the nurses felt that they were less sceptic towards the vaccine.

”came back to vaccinate after having watched the film” [nurse, #1]
“some become softer, the film is really helpful actually” [nurse, #4]

According to the nurses, the parents believed the film was a valuable tool because it included relevant evidence-based information and highlighted the perspective of the parents. Moreover, the film was perceived as credible as most of the experts and parents participating in the film were of Somali origin and spoke Somali. The experts were also well-known, trusted, and respected authorities in the community.

Nurses expressed that they perceived parents to be less questioning and more open to discussions related to MMR vaccination after the interventions. Parents had become more open to receiving information and having a dialogue. Moreover, the nurses also felt that parents had gained more knowledge, in particular about autism and posed fewer questions about the disease.

”[it is] difficult to measure, it is just a feeling I have” [nurse, #1]
“[I] believe that there are more who are knowledgeable about autism, actually” [nurse, #8]

In addition, the card and the film were especially useful tools during the outbreak of measles after the intervention, as expressed by this nurse:

“During the outbreak we tried to reach out in every way by using information cards, the film, visit to open day care, pediatricians informing…” [nurse, #7]

Discussion

Findings from the present study suggest that tailored interventions with culturally tailored materials can facilitate nurses’ communication about vaccinations with parents of Somali descent having low vaccine acceptance. From the perspective of the nurses’, the tailored intervention using diverse tools supported the dialogue, dispelled myth and reduced language barriers while also giving nurses cultural insights. Importantly, nurses gained additional confidence in the scientific evidence supporting that MMR vaccination does not cause autism and they were now able to dispel the myth in their dialogue and better convey this information to the parents. The seminars both provided and reinforced knowledge that boosted the nurses' confidence in discussing autism which made them feel more secure when communicating with the parents. Following the intervention and by using the tailored tools provided, the nurses engaged in discussions regarding MMR vaccinations and autism with parents in a different way. Before the intervention, some nurses hesitated themselves and did not address parents when having concerns regarding MMR vaccinations [13]. In addition, tools were also useful during the outbreak of measles in 2017.

Before the intervention was implemented, key barriers were parents fearing that their children would “stop talking” following an MMR vaccination and some parents also described unpleasant encounters with nurses [12, 13]. On the other hand, a key facilitator for vaccinating parents in the community was their trust in nurses [12, 13]. Autism was a sensitive topic that both parents and nurses sidestepped when discussing MMR vaccinations. Similar barriers to vaccination due to fear of autism has also been observed in a Somali community in the US [18, 19].

Factors influencing sub-optimal, vaccination coverage are context specific, complex, and multidimensional [27]. Factors affecting declining vaccine acceptance include barriers in availability and convenience in healthcare systems as well as social determinants, social and cultural norms [28]. In addition, these factors are often specific for their context, setting, type of vaccine and they may also change over time [27]. Limited access to health care and vaccination services for populations has also influenced sub-optimal vaccination coverage in Europe [29].

Using the TIP framework for this intervention project was useful for gaining a thorough understanding of the barriers and drivers for MMR vaccination in the Somali community [24]. Previous studies have

For peer review only - http://bmjopen.bmj.com/site/about/guidelines.xhtml

highlighted the crucial role of nurses and healthcare providers in parental vaccine acceptance, trust, and decision-making [30-33]. The belief and attitudes of healthcare workers are essential to parental vaccine acceptance and the demand for childhood vaccinations [34, 35]. Hence, it was deemed necessary to tailor interventions for the nurses specifically as part of the multi-component intervention project and to complement the interventions targeting the parents. Multi-component, dialogue-based, and tailored interventions appear to be more effective [36]. In addition, an important aspect of the Swedish national immunization program is the Child Healthcare Services structure and its responsibility for providing and implementing childhood vaccinations [37]. Parents often meet the same nurses for the child's health and development checkups as well as for vaccinations and therefore the nurses have a crucial role in the success of the vaccination program. Although our present study suggests improvements in the dialogue from the perspective of the nurses, the nurses will still be faced with recurring challenges and parents having questions and concerns regarding vaccinations. Hence, continued work and effort are needed to support the positive trend in this community. The current study assessed the dialogue and situation 2 years after the implementation of the intervention. Further evaluations of long-term outcomes are needed as well as an assessment of the vaccination coverage in the community over time. As there are workforce turnover, it would be of importance that newly recruited nurses in the community are offered similar training and introduction to the tools. The current intervention was implemented in 2015-2017, repeating the seminars and reinforcing the information for all nurses may also be important for continued capacity building and sustainability. Generally, vaccinating nurses could benefit from training and boosting of knowledge routinely regarding communication with parents about vaccinations.

Evaluations of tailored interventions are needed to increase the evidence base for effective interventions that can be used by public health authorities to not only increase vaccination acceptance or uptake but also contribute to resilience for national immunization programs. By addressing pockets with suboptimal vaccination coverage and identifying the facilitators and barriers to vaccination, tailored interventions can be designed and implemented to improve vaccination uptake and health equity.

Methodological considerations
Throughout the research process, trustworthiness has been strengthened by several activities. Several members of the interdisciplinary research team had expertise in qualitative methodology and were also familiar with the setting, community, and culture over an extended period before the intervention was implemented. Moreover, the research team familiarized themselves with the data initially and regularly discussed the analysis to strengthen its validity. This study is limited, however, as it only focused on the perspective of the nurses, further studies are needed to capture the perspective of the parents. Although the number of participants in this interview study is small the results are relevant for this specific community and setting, generalizing the findings to other settings should be done with caution.

Conclusion
Nurses' perceived the dialogue to be facilitated and enhanced with parents' vaccination concerns, following the implementation of tailored interventions. Particularly, nurses increased confidence in conveying evidence-based information about autism and MMR vaccination and felt better equipped to convey this information to the parents and to dispel the myth linking autism and MMR in their dialogue with the parents. In addition, the tailored tools were also perceived to reduce language barriers. Child health nurses have a crucial role in the vaccine acceptance and uptake in the Swedish national immunization program and interventions aiming to strengthen the nurses in their communication and dialogue with parents are therefore important. Further studies are needed for assessing the long-term outcomes of the multi-component intervention as well as vaccination coverage within the studied community.

Acknowledgements

For peer review only - http://bmjopen.bmj.com/site/about/guidelines.xhtml

The authors wish to thank the Stockholm Region child health services for their support and collaboration in this study. A sincere thank you to all the nurses at the child health care centers who kindly participated with openness and commitment.

Contributors

EA, AJ, AK, AL and KGR designed the study. All authors contributed to the analysis and interpretation of data. EA, AJ and KGR drafted the first version of this paper and AK, AL and AR have revised the paper critically and substantially. The final version of the paper has been approved by all authors and they all agree to be accountable for all aspects of the work.

Funding

The current study was funded by The Public Health Agency of Sweden.

Competing interests

The authors declare no conflict of interest.

Ethical approval

The study received ethical approval for the interviews from the Regional Ethics Committee in Stockholm, Sweden, Dnr 2016/1518-31/5.

Data availability statement

No additional data available.

For peer review only

References

1. European Centre for Disease Prevention and Control. Measles and rubella surveillance – 2017. Stockholm: ECDC; 2018.
2. World Health Organization. Measles emergency in the European Region: WHO; 2019 [Available from: https://www.who.int/europe/emergencies/situations/measles-emergency-in-the-european-region.
3. World Health Organization. Tailoring Immunization Programmes (TIP). Copenhagen: WHO Regional Office for Europe; 2019.
4. Butler R, MacDonald NE. Diagnosing the determinants of vaccine hesitancy in specific subgroups: The Guide to Tailoring Immunization Programmes (TIP). Vaccine. 2015.
5. Habersaat KB, Jackson C. Understanding vaccine acceptance and demand—and ways to increase them. Bundesgesundheitsblatt - Gesundheitsforschung - Gesundheitsschutz. 2019.
6. Folkhälsomyndigheten. Barnvaccinationsprogrammet i Sverige 2020 - Årsrapport. 2021.
7. Folkhälsomyndigheten. Barnvaccinationsprogrammet i Sverige 2019 – Årsrapport. 2020.
8. World Health Organization. SIXTH MEETING OF THE EUROPEAN REGIONAL VERIFICATION COMMISSION FOR MEASLES AND RUBELLA ELIMINATION (RVC). 2017.
9. Folkhälsomyndigheten. Barriers and motivating factors to MMR vaccination in communities with low coverage in Sweden. 2015.
10. Folkhälsomyndigheten. Barnvaccinationsprogrammet i Sverige 2017. 2018.
11. Bystrom E, Lindstrand A, Likhite N, Butler R, Emmelin M. Parental attitudes and decision-making regarding MMR vaccination in an anthroposophic community in Sweden--a qualitative study. Vaccine. 2014;32(50):6752-7.
12. Jama A, Ali M, Lindstrand A, Butler R, Kulane A. Perspectives on the Measles, Mumps and Rubella Vaccination among Somali Mothers in Stockholm. International journal of environmental research and public health. 2018;15(11).
13. Jama A, Lindstrand A, Ali M, Butler R, Kulane A. Nurses' Perceptions Of MMR Vaccine Hesitancy In An Area With Low Vaccination Coverage. Pediatric health, medicine and therapeutics. 2019;10:177-82.
14. Godoy-Ramirez K, Byström E, Lindstrand A, Butler R, Ascher H, Kulane A. Exploring childhood immunization among undocumented migrants in Sweden - following qualitative study and the World Health Organizations Guide to Tailoring Immunization Programmes (TIP). Public health. 2019;171:97-105.
15. Wakefield AJ, Murch SH, Anthony A, Linnell J, Casson DM, Malik M, et al. Ileal-lymphoid-nodular hyperplasia, non-specific colitis, and pervasive developmental disorder in children. Lancet. 1998;351(9103):637-41.
16. Taylor LE, Swerdfeger AL, Eslick GD. Vaccines are not associated with autism: an evidence-based meta-analysis of case-control and cohort studies. Vaccine. 2014;32(29):3623-9.
17. Madsen KM, Hviid A, Vestergaard M, Schendel D, Wohlfahrt J, Thorsen P, et al. A population-based study of measles, mumps, and rubella vaccination and autism. The New England journal of medicine. 2002;347(19):1477-82.
18. Bahta L. Addressing MMR Vaccine Resistance in Minnesota's Somali Community. MINNESOTA MEDICINE. 2015.
19. Gahr P, DeVries AS, Wallace G, Miller C, Kenyon C, Sweet K, et al. An Outbreak of Measles in an Undervaccinated Community. Pediatrics. 2014;134(1):e220-e8.
20. Paterson P, Meurice F, Stanberry LR, Glismann S, Rosenthal SL, Larson HJ. Vaccine hesitancy and healthcare providers. Vaccine. 2016;34(52):6700-6.
21. Ames HM, Glenton C, Lewin S. Parents' and informal caregivers' views and experiences of communication about routine childhood vaccination: a synthesis of qualitative evidence. Cochrane Database Syst Rev. 2017;2(2):Cd011787.
22. Berry NJ, Danchin M, Trevena L, Witteman HO, Kinnersley P, Snelling T, et al. Sharing knowledge about immunisation (SKAI): An exploration of parents' communication needs to inform development of a clinical communication support intervention. Vaccine. 2018;36(44):6480-90.

For peer review only - http://bmjopen.bmj.com/site/about/guidelines.xhtml

23. Karafillakis E, Dinca I, Apfel F, Cecconi S, Wűrz A, Takacs J, et al. Vaccine hesitancy among healthcare workers in Europe: A qualitative study. Vaccine. 2016;34(41):5013-20.
24. Jama A, Appelqvist E, Kulane A, Karregård S, Rubin J, Nejat S, et al. Design and implementation of tailored intervention to increase vaccine acceptance in a Somali community in Stockholm, Sweden - based on the Tailoring Immunization Programmes approach. Public Health in Practice. 2022;4:100305.
25. Folkhälsomyndigheten. Informationsmaterial om vaccinationer på somaliska 2022 [Available from: https://www.folkhalsomyndigheten.se/smittskydd-beredskap/vaccinationer/informationsmaterial-om-vaccinationer/informationsmaterial-om-vaccinationer-pa-somaliska/.
26. Braun V, Clarke V. Using thematic analysis in psychology. Qualitative Research in Psychology. 2006;3(2):77-101.
27. MacDonald NE. Vaccine hesitancy: Definition, scope and determinants. Vaccine. 2015;33(34):4161-4.
28. Dubé E, Gagnon D, MacDonald N, Bocquier A, Peretti-Watel P, Verger P. Underlying factors impacting vaccine hesitancy in high income countries: a review of qualitative studies. Expert Review of Vaccines. 2018;17(11):989-1004.
29. Boyce T, Gudorf A, de Kat C, Muscat M, Butler R, Habersaat KB. Towards equity in immunisation. Euro Surveill. 2019;24(2).
30. Leask J, Kinnersley P, Jackson C, Cheater F, Bedford H, Rowles G. Communicating with parents about vaccination: a framework for health professionals. BMC pediatrics. 2012;12:154.
31. Gust DA, Darling N, Kennedy A, Schwartz B. Parents with doubts about vaccines: which vaccines and reasons why. Pediatrics. 2008;122(4):718-25.
32. Glanz JM, Wagner NM, Narwaney KJ, Shoup JA, McClure DL, McCormick EV, et al. A mixed methods study of parental vaccine decision making and parent-provider trust. Academic pediatrics. 2013;13(5):481-8.
33. Cooper LZ, Larson HJ, Katz SL. Protecting public trust in immunization. Pediatrics. 2008;122(1):149-53.
34. Simone B, Carrillo-Santisteve P, Lopalco PL. Healthcare workers role in keeping MMR vaccination uptake high in Europe: a review of evidence. Euro surveillance : bulletin europeen sur les maladies transmissibles = European communicable disease bulletin. 2012;17(26):20206.
35. Dube E. Addressing vaccine hesitancy: the crucial role of healthcare providers. Clin Microbiol Infect. 2017;23(5):279-80.
36. Dubé E, Gagnon D, MacDonald NE. Strategies intended to address vaccine hesitancy: Review of published reviews. Vaccine. 2015;33(34):4191-203.
37. Wettergren B, Blennow M, Hjern A, Söder O, Ludvigsson JF. Child Health Systems in Sweden. The Journal of Pediatrics. 2016;177:S187-S202.

For peer review only

Folkhälsomyndigheten
PUBLIC HEALTH AGENCY OF SWEDEN

TIP Sweden: Evaluation of tailored communication on vaccination in Rinkeby and Tensta, Stockholm

Interview Guide – Nurses at the Child Health Clinics

Aim: To investigate how the implemented interventions are experienced and received by CHC nurses and how the interventions have influenced their work towards vaccine-hesitant parents.

The interview

- Give a brief introduction on the purpose of the interview and procedures, as a follow-up to previous written information
- Consent to participate in the interview, recording, etc.

Date (yy/mm/dd):____/____/____

Code no. for the interview:

Place for the interview: ____________

Starting time: __:____ End: ___:____

Interviever: ____

Background information

- Employment title
- How long have you worked within the CHC/services?
- District you are working in (Rinkeby or Tensta)?
- How long have you worked in the district?

Your experience about the tailored interventions organized by the PHAS

- A series of three seminars was given during autumn in 2015, did you participate?
- Can you describe what you thought about the content of the seminars? What was good/less good?
- Did the seminars give you new knowledge? (About autism, cultural communication, summary about Wakefields research, diseases and the global situation?)
- Can you describe the discussions that followed the seminars?
- Have you been able to use the information from the lectures when you meet vaccine-hesitant parents? (Can you give an example of a recent conversation with a VH parent? what happened, what did they say, what was the outcome etc. How did they use the training? Perhaps ask for an example where the conversation went well, and one where it did not. ?)
- Would you like the seminars to be given again? (In what format? Themes?)
- Can you describe other interventions/competence enhancement activities related to vaccine hesitancy organized within the CHS? (What do you think about these? Have they been in useful, how?)

Solna Folkhälsomyndigheten, SE-171 82 Solna. Visit: Nobels väg 18. **Östersund** Folkhälsomyndigheten, SE-831 40 Östersund. Visit: Forskarens väg 3.
Telephone + 46 10 205 20 00 **E-mail** info@folkhalsomyndigheten.se **www.folkhalsomyndigheten.se**

For peer review only

Sida
2 (2)

Benefits from interventions by PHAS in your daily work

- Are you familiar with the other intervention activities within the project, targeting the Somali speaking parents? (What do you think about them? Somali film, seminars, peers, postcard)
- Have you had any use of the interventions, generally? Can you describe if / how you have benefited from these in your work? (Referred to the parents? Used for discussion))
- Do you know about the peer group created within the project? (Have you met anyone in the group? Collaborated in any way? Have they served as support in your work)

Changed mode of work following the intervention

- Has your mode of work changed or been affected since the time before the interventions started? (In what way? Regarding autism?)
- Do you as nurses work differently at the CHC when approaching vaccine-hesitant parents? (Can you describe how? Internal communication? Autism?)
- Can you identify other / new groups of parents in the neighborhood who are hesitant towards vaccinations?

Your perception on parents' experiences of the interventions and change in parents' knowledge

- How do you think that Somali speaking parents experience the interventions? (Have they been reached? Their attitude towards the activities, seminars, films, peers)
- What questions and concerns do the parents have today? (How do they reason?)
- Do you experience any differences in VH parent´s knowledge compared with the time before the intervention?
- Do you experience any differences in attitudes among vaccine-hesitant parents compared with the time before the intervention? (What differences? What about their perception of social norms around vaccination in the community? (social opportunity COM-B) Larger or minor problem?)
- How do parents reason about autism today? (Any changes? How?)
- Do you experience a difference in behavior (decision to vaccinate) among vaccine-hesitant parents as compared to how it was before the interventions? Do they perceive a difference in decision-making in parents (reflective versus automatic motivation COM-B)

Future perspectives

- Do you need any further support in specific questions/areas regarding vaccination? (Which areas? Format? Something you have missed? Other interventions?)
- Do you need further knowledge on specific issues related to vaccine-hesitancy? (Which? How would you like to have knowledge?)
- Do you have any suggestion on other ways to reach vaccine-hesitant parents (which the staff at the CHC can work with?
- Do you have any thoughts about the group of peers? (Any benefit from their work, suggestions for cooperation, joint activities)?

Before we finish the interview

- Is there anything else you would like to address or add that we have not yet discussed?

Thank you for your participation!

Solna Folkhälsomyndigheten, SE-171 82 Solna. Visit: Nobels väg 18. **Östersund** Folkhälsomyndigheten, SE-831 40 Östersund. Visit: Forskarens väg 3.
Telephone + 46 10 205 20 00 **E-mail** info@folkhalsomyndigheten.se **www.folkhalsomyndigheten.se**

For peer review only

Standards for Reporting Qualitative Research (SRQR)*

http://www.equator-network.org/reporting-guidelines/srqr/

Page/line no(s).

Title and abstract

**Title** - Concise description of the nature and topic of the study Identifying the study as qualitative or indicating the approach (e.g., ethnography, grounded theory) or data collection methods (e.g., interview, focus group) is recommended	Page 1
**Abstract** - Summary of key elements of the study using the abstract format of the intended publication; typically includes background, purpose, methods, results, and conclusions	Page 1

Introduction

**Problem formulation** - Description and significance of the problem/phenomenon studied; review of relevant theory and empirical work; problem statement	Introduction, page 3-4
**Purpose or research question** - Purpose of the study and specific objectives or questions	End of introduction, page 4

Methods

**Qualitative approach and research paradigm** - Qualitative approach (e.g., ethnography, grounded theory, case study, phenomenology, narrative research) and guiding theory if appropriate; identifying the research paradigm (e.g., postpositivist, constructivist/ interpretivist) is also recommended; rationale**	Methods, page 5-6
**Researcher characteristics and reflexivity** - Researchers' characteristics that may influence the research, including personal attributes, qualifications/experience, relationship with participants, assumptions, and/or presuppositions; potential or actual interaction between researchers' characteristics and the research questions, approach, methods, results, and/or transferability	Methods & methodological considerations, page 5-6 & 10
**Context** - Setting/site and salient contextual factors; rationale**	Methods, page 5-6
**Sampling strategy** - How and why research participants, documents, or events were selected; criteria for deciding when no further sampling was necessary (e.g., sampling saturation); rationale**	Methods, page 5-6
**Ethical issues pertaining to human subjects** - Documentation of approval by an appropriate ethics review board and participant consent, or explanation for lack thereof; other confidentiality and data security issues	Methods, page 6
**Data collection methods** - Types of data collected; details of data collection procedures including (as appropriate) start and stop dates of data collection and analysis, iterative process, triangulation of sources/methods, and modification of procedures in response to evolving study findings; rationale**	Methods, page 5-6
**Data collection instruments and technologies** - Description of instruments (e.g., interview guides, questionnaires) and devices (e.g., audio recorders) used for data collection; if/how the instrument(s) changed over the course of the study	Methods, page 5-6 and also interview guide in appendix
**Units of study** - Number and relevant characteristics of participants, documents, or events included in the study; level of participation (could be reported in results)	Methods, page 5-6

For peer review only - http://bmjopen.bmj.com/site/about/guidelines.xhtml

For peer review only

**Data processing** - Methods for processing data prior to and during analysis, including transcription, data entry, data management and security, verification of data integrity, data coding, and anonymization/de-identification of excerpts	Methods, page 5-6
**Data analysis** - Process by which inferences, themes, etc., were identified and developed, including the researchers involved in data analysis; usually references a specific paradigm or approach; rationale**	Methods, page 5-6
**Techniques to enhance trustworthiness** - Techniques to enhance trustworthiness and credibility of data analysis (e.g., member checking, audit trail, triangulation); rationale**	Methodological considerations, page 10

Results/findings

**Synthesis and interpretation** - Main findings (e.g., interpretations, inferences, and themes); might include development of a theory or model, or integration with prior research or theory	Results, pages 6-9
**Links to empirical data** - Evidence (e.g., quotes, field notes, text excerpts, photographs) to substantiate analytic findings	Quotes included throughout the in results section, pages 6-9

Discussion

**Integration with prior work, implications, transferability, and contribution(s) to the field -** Short summary of main findings; explanation of how findings and conclusions connect to, support, elaborate on, or challenge conclusions of earlier scholarship; discussion of scope of application/generalizability; identification of unique contribution(s) to scholarship in a discipline or field	Pages 9-10
**Limitations** - Trustworthiness and limitations of findings	Methodological considerations, page 10

Other

**Conflicts of interest** - Potential sources of influence or perceived influence on study conduct and conclusions; how these were managed	See statement, page 11
**Funding** - Sources of funding and other support; role of funders in data collection, interpretation, and reporting	See statement, page 10

*The authors created the SRQR by searching the literature to identify guidelines, reporting standards, and critical appraisal criteria for qualitative research; reviewing the reference lists of retrieved sources; and contacting experts to gain feedback. The SRQR aims to improve the transparency of all aspects of qualitative research by providing clear standards for reporting qualitative research.
**The rationale should briefly discuss the justification for choosing that theory, approach, method, or technique rather than other options available, the assumptions and limitations implicit in those choices, and how those choices influence study conclusions and transferability. As appropriate, the rationale for several items might be discussed together.

Reference:
O'Brien BC, Harris IB, Beckman TJ, Reed DA, Cook DA. **Standards for reporting qualitative research: a synthesis of recommendations.** *Academic Medicine*, Vol. 89, No. 9 / Sept 2014 DOI: 10.1097/ACM.0000000000000388

For peer review only - http://bmjopen.bmj.com/site/about/guidelines.xhtml
